# Molecular Basis of Oncogenic PI3K Proteins

**DOI:** 10.3390/cancers17010077

**Published:** 2024-12-30

**Authors:** Zhi Sheng, Patrick Beck, Maegan Gabby, Semhar Habte-Mariam, Katherine Mitkos

**Affiliations:** 1 Fralin Biomedical Research Institute at VTC, Roanoke, VA 24016, USA; 2 Department of Internal Medicine, Virginia Tech Carilion School of Medicine, Roanoke, VA 24016, USA; 3 Department of Neurosurgery, Virginia Tech Carilion School of Medicine, Roanoke, VA 24016, USA; 4 School of Neuroscience, Virginia Polytechnic Institute and State University, Blacksburg, VA 24061, USA; 5 Faculty of Health Science, Virginia Polytechnic Institute and State University, Blacksburg, VA 24061, USA; 6 Division of General Pediatrics, Children’s Hospital of Philadelphia, Philadelphia, PA 19104, USA

**Keywords:** PI3K, protein structure, oncogenic transformation, oncogenic mutation

## Abstract

The dysregulation of phosphatidylinositol 3-kinase (PI3K) signaling plays a pivotal role in driving neoplastic transformation by promoting uncontrolled cell survival and proliferation. This oncogenic activity is primarily caused by mutations that are frequently found in PI3K genes and constitutively activate the PI3K signaling pathway. However, tumorigenesis can also arise from nonmutated PI3K proteins adopting unique active conformations, further complicating the understanding of PI3K-driven cancers. Recent structural studies have illuminated the functional divergence among highly homologous PI3K proteins, revealing how subtle structural alterations significantly impact their activity and contribute to tumorigenesis. In this review, we summarize current knowledge of Class I PI3K proteins and aim to unravel the complex mechanism underlying their oncogenic traits. These insights will not only enhance our understanding of PI3K-mediated oncogenesis but also pave the way for the design of novel PI3K-based therapies to combat cancers driven by this signaling pathway.

## Simple Summary

In many cancers, the phosphatidylinositol 3-kinase (PI3K) signaling pathway is persistently activated due to genetic mutations, while certain wild-type PI3K proteins adopt active conformations that further enhance the pathway’s oncogenic potential. Recent studies have uncovered differences in the three-dimensional structures of homologous PI3K proteins, underscoring how subtle variations contribute to their functional divergence and tumorigenic activity. Understanding these structural and functional nuances is essential to elucidate the molecular basis driving PI3K signaling in cancer and to develop targeted therapeutic strategies.

## 1. Introduction

**Figure 1:**
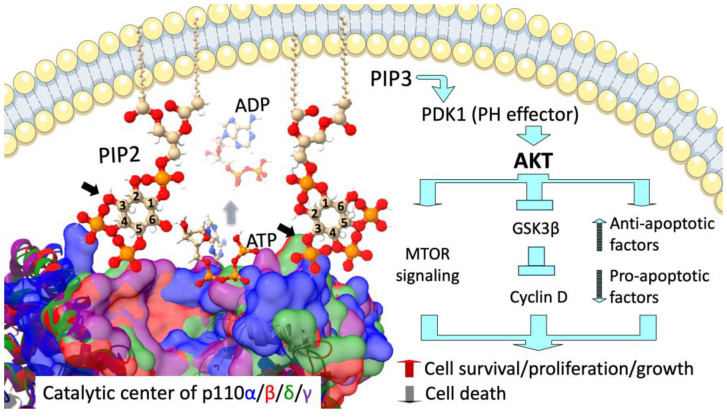
Lipid phosphorylation by PI3K kinases and its consequences. At the catalytic center of the Class I PI3K kinases—p110α (blue), p110β (red), p110δ (green), or p110γ (purple)—the γ-phosphate from ATP is transferred to the D3 position of the inositol ring of PIP2 (arrow), yielding PIP3 and ADP. PIP3 subsequently recruits and activates the PH effector PDK1, leading to the activation of AKT. Once activated, AKT changes the following cellular processes: (1) Stimulating cell proliferation via activating MTOR; (2) Promoting cell growth by inactivating glycogen synthase kinase 3β (GSK3β) to release its inhibition of Cyclin D; (3) Suppressing cell death via enhancing the activity of anti-apoptotic factors (e.g., MCL1/BCL2) while inhibiting pro-apoptotic proteins (e.g., BH3-only proteins). The activation of cell survival, proliferation, and growth by PI3K activation ultimately contributes to oncogenic transformation. The structural models of human PI3K kinases were predicted using AlphaFold. The structures of PIP2, PIP3, ADP, and ATP were retrieved from PubChem. All 3D structures were reconstructed using ChimeraX-1.8, and the catalytic centers of the four Class I kinases were aligned using ChimeraX-1.8 Matchmaker.

The phosphatidylinositol 3-kinase (PI3K) pathway, discovered in the 1980s, has been a focal point of cell biology owing to its critical role in cell survival, death, and metabolism. This pathway is vital for early tissue and organ development and is implicated in the pathogenesis of various human diseases, including cancer. The primary upstream activators of PI3K are receptor tyrosine kinases (RTKs) and G protein-coupled receptors (GPCRs), which either activate PI3K directly or signal through Ras or Rho family GTPases [1,2,3,4,5]. As illustrated in Figure 1, upon activation, PI3K kinases along with their adapter proteins are recruited to the inner layer of the cellular membrane, where they catalyze the transfer of a phosphate group from adenosine triphosphate (ATP) to the D3 position of the inositol ring in phosphatidylinositol 4,5-bisphosphate (PIP2 or PtdIns 4,5-P). This reaction yields phosphatidylinositol 3,4,5-trisphosphate (PIP3 or PtdIns 3,4,5-P) and adenosine diphosphate (ADP). PIP3 then serves as a secondary messenger that recruits and activates the effector kinase pyruvate dehydrogenase kinase 1 (PDK1), harboring the pleckstrin homology (PH) domain that has high affinity for PIP3. This subsequently results in the activation of protein kinase B (AKT), the major effector kinase downstream of PI3K that initiates global changes in gene expression that promote cell survival while suppressing cell death [6]. Phosphatase and tensin homolog (PTEN) counteracts PI3K activity by dephosphorylating PIP3 into PIP2, thereby acting as an inhibitor of PI3K signaling pathway. While AKT is primarily activated via PI3K, certain signaling pathways also converge onto the activation of AKT. For example, AMP-activated protein kinase (AMPK), a central regulator of metabolic homeostasis, can activate AKT, contributing to the roles of AKT in tumorigenesis and therapy resistance [7,8]. Although PI3K upstream activators and downstream effectors as well as these alternative pathways are critical, they are beyond the scope of this discussion and have been reviewed previously [9,10,11,12]. This review focuses on understanding the central mechanism of PI3K kinases and their adaptor proteins in tumorigenesis.

There are three major classes of PI3K genes, initially proposed by Domin and Waterfield [13]: Class I, II, and III. These classes are categorized based upon their lipid substrate specificity. The Class I PI3K targets PIP2, whereas the Class II PI3K acts on phosphatidylinositol (PI or Ptdlns) and phosphatidylinositol 4-phosphate (PI4P or PtdIns4P), and the Class PI3K III specifically phosphorylates PI4P [1,6,14]. Among all PI3K genes, PI3K catalytic subunit type 3 (PIK3C3, also known as Vps34) in the Class III family was the first to be assigned a function—regulating intracellular protein trafficking in yeast [14]. The biology of Class II/III families has been reviewed previously [15,16]. This review focuses exclusively on Class I PI3K signaling which comprises the IA and IB subfamilies. Class I PI3K genes were first identified in the 1980s by Lewis Cantley’s group [17]. Since then, efforts on purification, cloning and genomic characterization have revealed divergent heterodimeric signaling complexes consisting of four catalytic subunits (termed kinases hereafter based on their function) and six regulatory subunits including two splicing variants (termed adaptors hereafter) in the Class I family [18,19,20,21,22,23,24,25,26,27,28,29]. The IA subfamily includes three kinases—p110α, p110β, and p110δ—encoded by the genes phosphatidylinositol-4,5-bisphosphate 3-kinase catalytic subunit α, β, and δ (PIK3CA, PIK3CB, and PIK3CD), respectively. These kinases pair with three adaptors—p85α, p85β, or p55γ—encoded by PI3K regulatory subunit 1, 2, or 3 (PIK3R1, PIK3R2, or PIK3R3), respectively [30]. Additionally, p85α has two splicing variants, p55α and p50α [31,32]. Each kinase binds to a single adaptor, forming an obligate heterodimer [33]; however, there is no evidence of preferential binding between specific kinases and adaptors, despite structural and functional differences among PI3K proteins [23]. The IB subfamily is composed of one kinase, p110γ, encoded by phosphatidylinositol-4,5-bisphosphate 3-kinase catalytic subunit γ (PIK3CG) and two adaptors: p101 and p84, encoded by PI3K regulatory subunit 5 (PIK3R5) and PI3K regulatory subunit 6 (PIK3R6), respectively [26,34]. Phosphorylation levels of both IA and IB kinases and/or adaptors are not considered as a reliable measure of in vivo PI3K activities because there is no correlation between the kinase activity of PI3K complexes and autophosphorylation or phosphorylation by other kinases [23,35,36,37]. To differentiate the single kinases (p110α, p110β, p110δ, or p110γ) from their complexes, this review refers to kinase/adaptor assemblies as PI3Kα, PI3Kβ, PI3Kδ, or PI3Kγ, respectively.

In vitro studies reveal that free monomers of recombinant p110 kinases chromatographically purified from insect cells can convert PIP2 into PIP3 in a cell free system without adaptors but remain unstable in solution [38,39]. When recombinant IA adaptors are added, stable heterodimeric kinase/adaptor complexes form, though their in vitro kinase activity is limited [38], likely due to adaptor-mediated stabilization and inhibition. Interestingly, this inhibition is not observed in the IB family, as p101 does not block the in vitro kinase activity of p110γ [26,40]. Nonetheless, activation of PI3K signaling in vivo requires adaptors because expressing p110 kinases alone in mammalian cells displays no kinase activity, and PI3K is only activated when both kinases and adaptors are present [20,22,39,41]. This is because the association of PI3K complexes with upstream RTKs or GPCRs is an essential step prior to PI3K activation. For instance, bisphosphopeptides containing the pYXXM motif mimic the domain in RTKs for association with PI3K adaptors. In in vitro experiments, pYXXM peptide usually binds to the purified PI3Kα/β/δ complexes and subsequently activates the recombinant p110 kinases, resembling the in vivo activation of PI3K [22,38]. Collectively, these biochemical findings suggest that, in mammalian cells, PI3K heterodimers are kept in an inhibited state in the cytosol and become activated upon translocation to the membrane [33]. This spatiotemporal change induces conformational shifts in PI3K protein assemblies, transitioning them from a locked, inactive conformation to an open, active conformation [1,42]. These early studies highlight the importance of three-dimensional (3D) structures in understanding PI3K regulation, particularly in the context of cancer. Recent structural studies have generated a wealth of information, broadening and deepening our knowledge regarding the molecular basis of oncogenic PI3K proteins. This review provides a comprehensive overview of PI3K protein structures and explores the complex roles of PI3K in oncogenesis.

## 2. Structural Details and Functional Determinants of PI3K Proteins

### 2.1. Domains in PI3K Proteins

**Figure 2:**
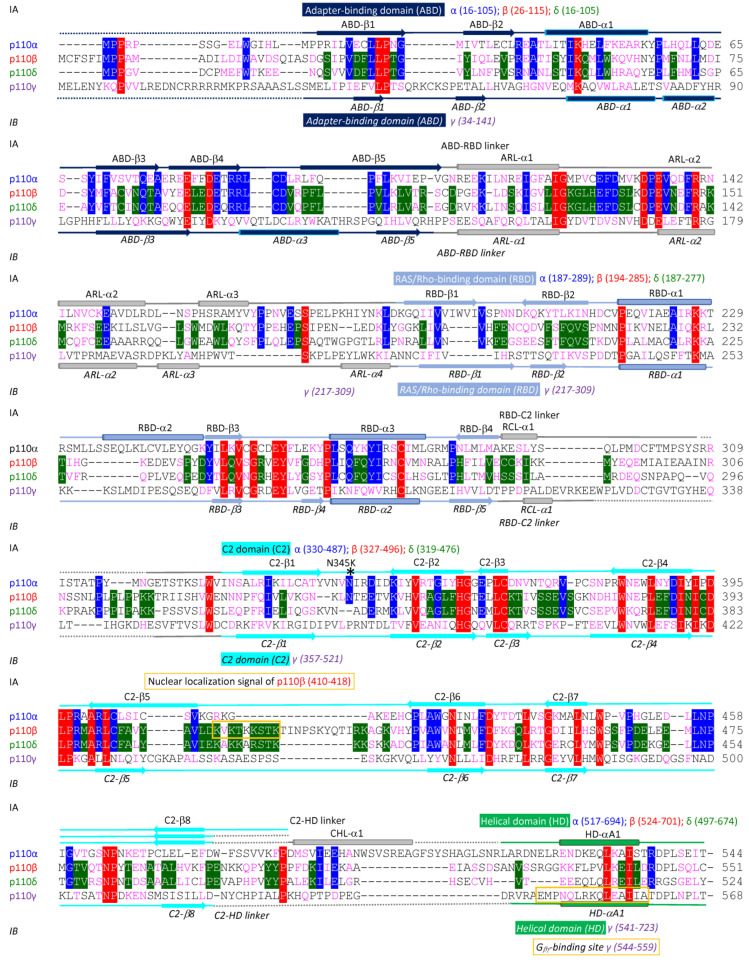

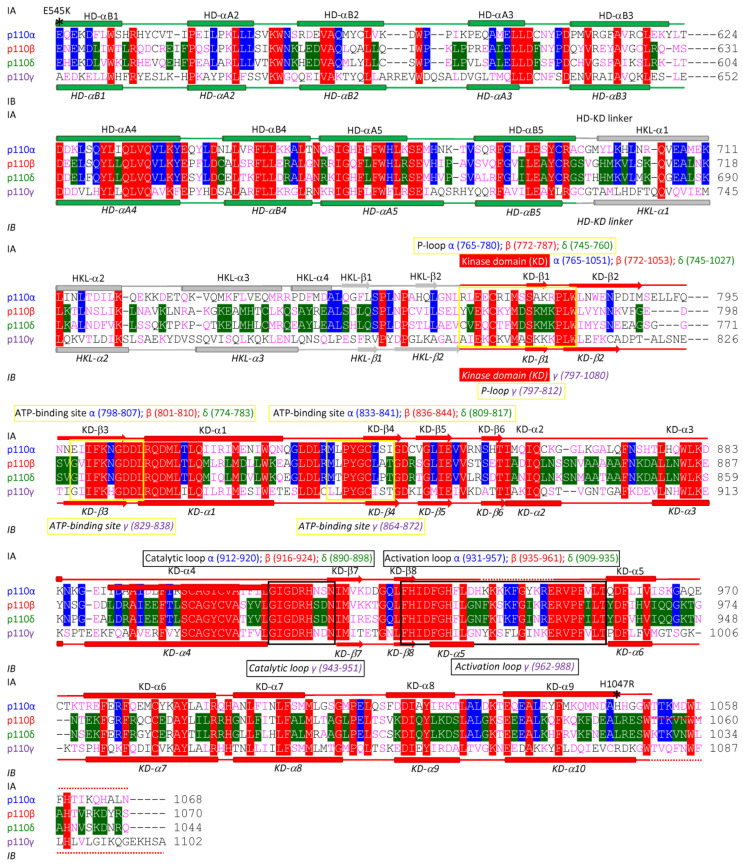
Alignment of Class I PI3K kinases. Protein sequences of PI3K kinases were obtained from UniProt: PIK3CA/p110α: P42336; PIK3CB/p110β: P42338; PIK3CD/p110δ: O00329; PIK3CG/p110γ: P48736. Sequence alignment was performed using Clustal Omega and COBALT. Structural and functional domains were annotated based on UniProt and published literature (PMID:10580505; 18633356; 21362552; 34452907). Functional domains are color-coded: ABD (dark blue), RBD (light blue), C2 (cyan), HD (green), KD (red), and Linker regions (grey). Domains and motifs in p110α (representing class IA) are shown on the top, while those in p110γ (IB) are shown on the bottom. Identical residues are highlighted in red (all p110 kinases), blue (class IA p110 kinases), or green (shared between p110β and p110δ). Conserved residues are shown in magenta. Structural elements such as β-sheets (arrows), α-helices (cylinders), and loops/turns (lines) are indicated with undetermined regions represented by dotted lines. Key motifs, including nuclear localization signal, ATP-binding sites, catalytic loop, and activation loop, are labeled. Oncogenic mutations are marked with an asterisk.

**Figure 3:**
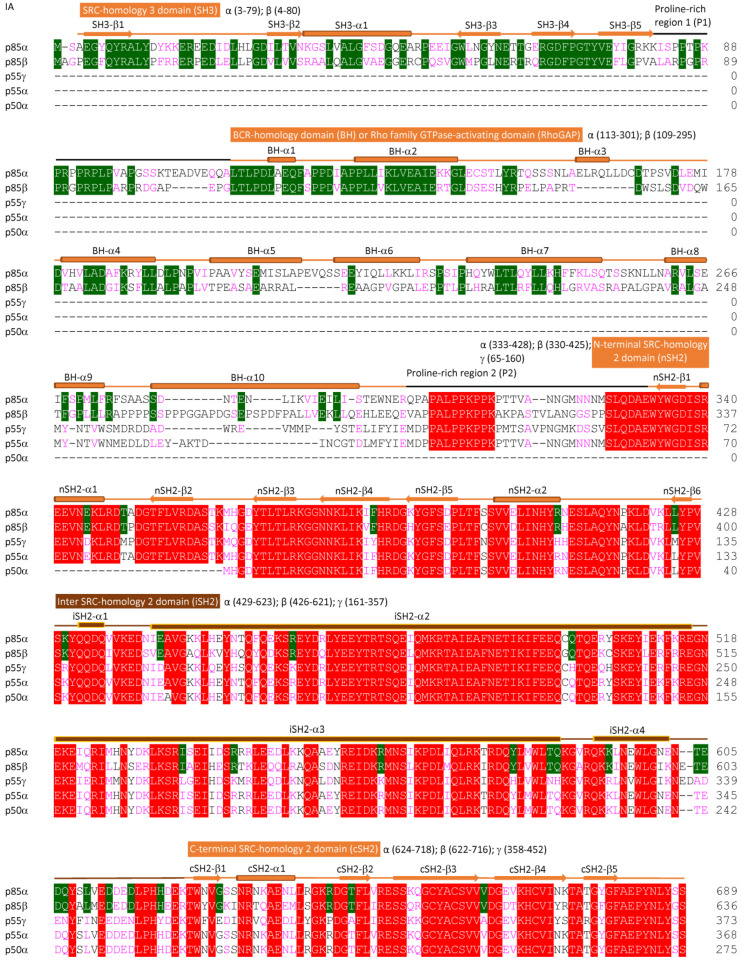

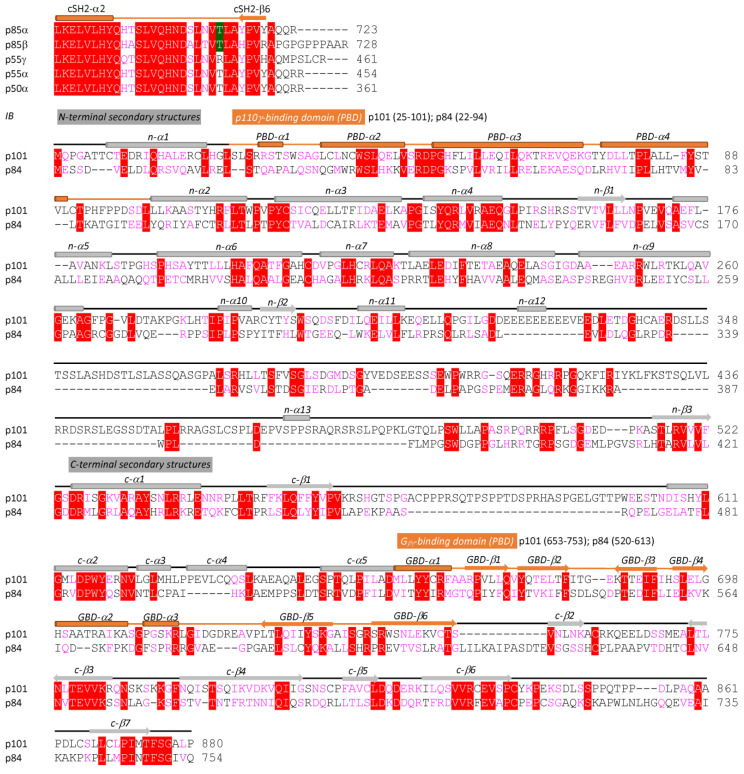
Alignment of Class I PI3K regulatory subunits. Protein sequences of PI3K regulatory subunits were obtained from UniProt: PIK3R1/p85α/p55α/p50α: P27986; PIK3R2/p85β: O00459; PIK3R3/p55γ: O92569; PIK3R5/p101: Q8WYR; PIK3R6/p84: Q5UE93. Sequence alignment was conducted using Clustal Omega and COBALT. Structural and functional domains were annotated based on Uniprot, AlphaFold-predicted 3D structures, and published literature (PMID:21362552 and 35429500). Functional domains in IA adapters are color-coded: SH3 (brown), P1/P2 (black), BH (brown), nSH2 (brown), iSH2 (dark brown), and cSH2 (brown). Functional domains in IB adapters are also color-coded: PBD/GBD (brown). Other N-terminal or C-terminal secondary structures are labeled in grey. Identical residues are highlighted in red among all Class IA adapters (p85α, p85β, p55γ, p55α and p50α) or between the Class IB adapters (p101 and p84). Identical residues are highlighted in green between p85α and p85β. Conserved residues among all adapters are in magenta. Structural elements are represented as follows: β-sheets (arrows), α-helices (cylinders), and loops/turns (lines).

**Figure 4:**
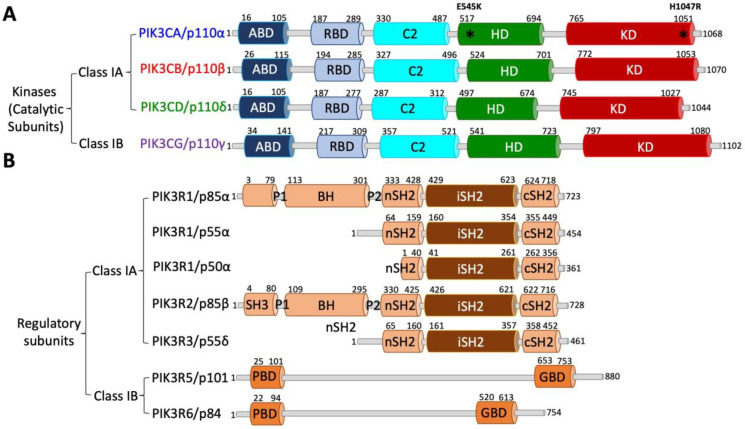
Functional domains in Class I PI3K proteins. (**A**) Illustration of functional domains in Class I PI3K kinases. (**B**) Illustration of functional domains in Class I PI3K adapters. Locations of domains are indicated. Linker regions are shown as grey bars. *: mutated residue.

The cloning of PI3K genes and purification of recombinant proteins have sparked the interest of many research groups in revealing the 3D conformations of PI3K complexes through extensive biochemical analyses, X-ray crystallography, nuclear magnetic resonance (NMR) spectroscopy, or the cutting-edge cryo-electron microscopy (cryo-EM) technique [18,25,35,39,41,43,44,45,46,47,48,49,50,51,52,53,54,55,56,57,58,59,60,61]. These studies have identified functional domains in both kinases and adaptors, as well as crucial roles of inter-subunit or intra-subunit protein–protein interactions in regulating PI3K activity. Class I kinases are composed of different numbers of amino acids (α: 1068, β: 1070, δ: 1044, and γ: 1102), as illustrated in Figure 2 and Figure 4A. Adaptors also have diverse compositions in amino acid sequences (p85α: 723, p85β: 728, p55γ: 461, p55α: 454, p50α: 361, p101: 880, and p84: 754), as summarized in Figure 3 and Figure 4B. Both Class IA and IB kinases contain five conserved functional domains (Figure 4A), which are, from N-terminus to C-terminus, the adapter-binding domain (ABD), Ras/Rho-binding domain (RBD), C2 domain (C2), helical domain (HD), and kinase domain (KD). Unlike kinases, Class IA and IB adaptors differ significantly in their protein sequences (Figure 3) and 3D functional structures (Figure 4B). Adaptors within the IA subclass also vary in their protein sizes due primarily to N-terminal deletions. For example, p85α and p85β shared similar amino acid compositions and domains; however, p55γ and the two p85α splicing forms, p55α and p50α, lack approximately 300–400 residues spanning several domains at the N-terminus (Figure 4B). IA adaptors p85α/p85β harbor the Src-Homology 3 domain (SH3), Proline-rich domain 1 (P1 or PR1), Breakpoint-Cluster-Region Homology domain (BH) or GTPase-activating protein domain (GAP), Proline-rich domain 2 (P2 or PR2), N-terminal Src-Homology 2 domain (nSH2), inter SH2 domain (iSH2), and C-terminal SH2 domain (cSH2). In contrast, other N-terminus-lacking IA adaptors only contain three SH2 domains. The 3D structures of p101 and p84 are largely unknown, with only the N-terminal p110γ-binding domain (PBD) and C-terminal Gβγ-binding domain (GBD) being identified recently [62].

To help readers visualize conformations of PI3K proteins, we have aligned PI3K proteins together based on their homology in amino acid sequences, secondary structures, and functional domains (Figure 2, Figure 3 and Figure 4).

### 2.2. Sequence Homology Among Full-Length PI3K Proteins

**Figure 5:**
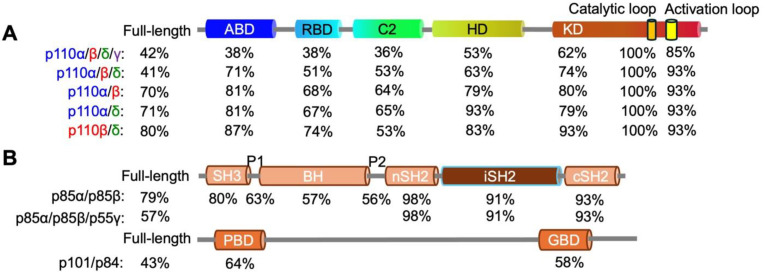
Homology among PI3K proteins. Using the sequence alignments shown in Figure 2 and Figure 3, homologous amino acids were identified, and the percentage of homology was calculated by dividing the number of homologous residues by the total number of residues in each PI3K kinases or adapters. Shown are homologies of full-length p110 kinase and their functional domains (**A**) and homologies of adapters and their functional domains (**B**).

By aligning the protein sequences of human PI3K kinases and adaptors, we analyzed the homologies among the four full-length kinases (Figure 5A) and the five full-length adaptors (Figure 5B). Approximately 40% of residues were homologous between Class IA p110α/β/δ and Class IB p110γ, as well as within the Class IA group. Notably, p110β shared 80% of residues with p110δ, while the homology between p110α and either p110β or p110δ was approximately 70%. These findings align with a previous report showing 40–55% homologous residues within Class IA kinases [63] and minimal homology between p110γ and the Class IA kinases [51]. As described above, Class IA and IB kinases bind to distinct sets of adaptors encoded by different genes. Consequently, there is no sequence homology between IA and IB adaptors (Figure 3 and Figure 5B), and these adaptors exhibit differential activities in regulating their kinase partners [22,26,38,51,62]. Within Class IA adapters, p85α and p85β showed 79% sequence similarity, indicating that these two isoforms are structurally similar. However, only 57% of analogous residues were found among p85α, p85β, and p55γ, primarily due to the N-terminal truncation in p55γ. In the Class IB subfamily, p101 and p84 shared only 43% of homologues residues. Despite these sequence differences, no significant functional divergence has been found among PI3K adapters.

### 2.3. 3D Structures of PI3K Kinases

**Figure 6:**
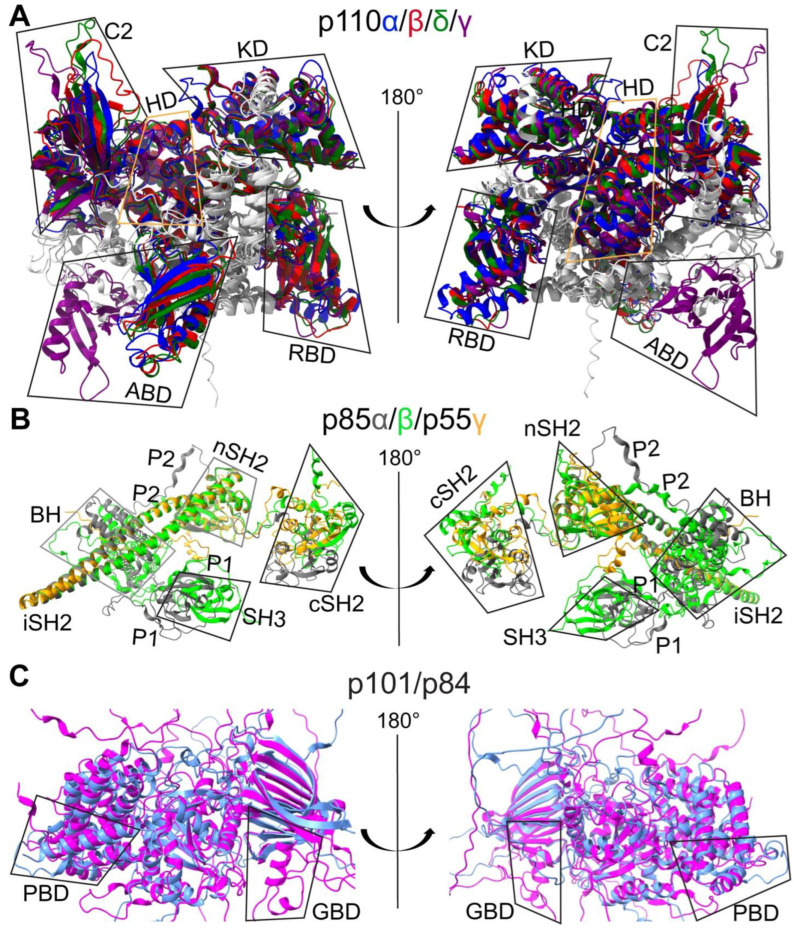
PI3K 3D structures. Since published crystal structures are often derived from truncated proteins or use non-human proteins, we employed AlphaFold to predict 3D conformations of full-length human PI3K proteins. These structures were aligned using ChimeraX-1.8 Matchmaker. (**A**) Aligned 3D structures of full-length PI3K kinases, which are color-coded as follows: p110α (blue), p110β (red), p110δ (green), p110γ (purple), and linker regions (light grey). (**B**) Aligned 3D structures of Class IA adaptors, which are color-coded as follows: p85α (grey), p85β (lime), and p55γ (gold). (**C**) Aligned 3D structures of Class IB adaptors, which are color-coded as follows: p101 (magenta) and p84 (light blue). Functional domains, as illustrated in Figure 2, Figure 3 and Figure 4, are labeled. The aligned structures are shown with a 180° rotation for a comprehensive view.

**Figure 7:**
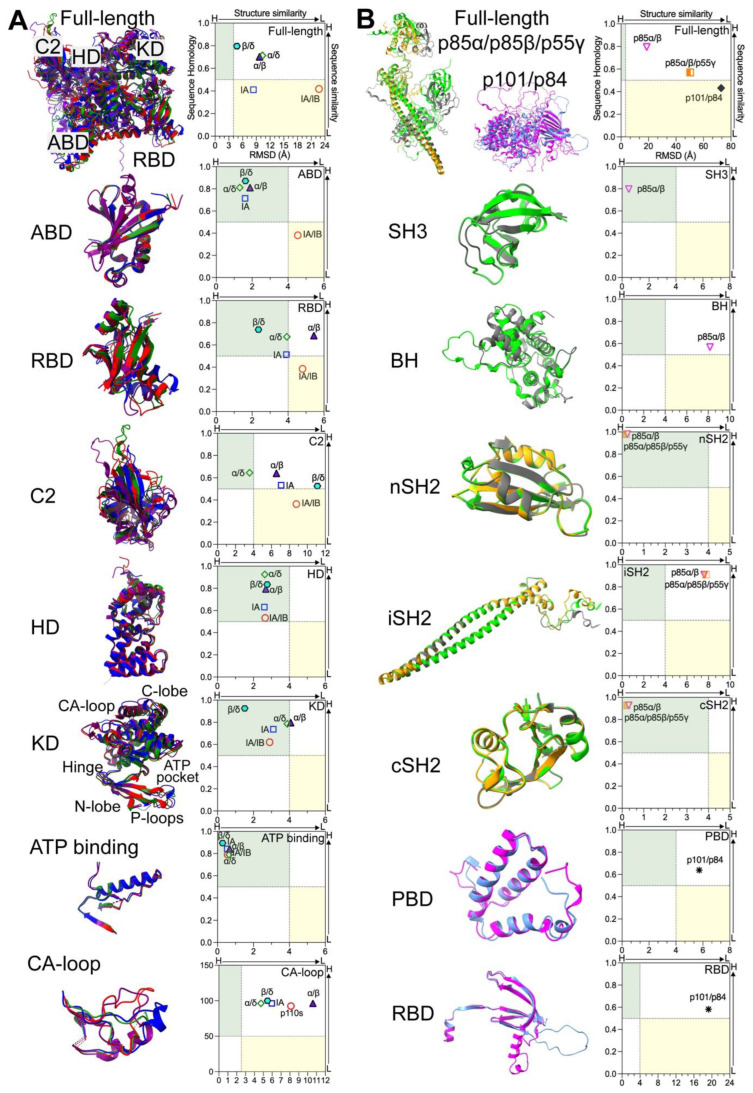
Structural homology of PI3K proteins and domains. RMSDs of full-length PI3K kinases or adapters, along with their functional domains, were determined using ChimeraX-1.8 Matchmaker. These values were plotted against percentages of homology shown in Figure 3. PI3K proteins are color-coded as follows: p110α (blue); p110β (red), p110δ (green); p110γ (purple); linker regions (light grey); p85α (grey); p85β (lime); p55γ (gold); p101 (magenta); and p84 (light blue). (**A**) Kinases and their domains/motifs. (**B**) Adaptors and their domains/motifs. Regions with high similarities (sequence homology > 50%/RMSD < 4Å) are highlighted in green, whereas low similarities (sequence homology < 50%/RMSDs > 4Å) are indicated in yellow. Red blank circles: Class IA kinases p110α/β/δ vs. IB kinase p110γ; Blue blank rectangles: within Class IA kinases p110α/β/δ; Purple filled triangles: p110α vs. p110β (α/β); Green blank diamonds: p110α vs. p110δ (α/δ); Cyan filled hexagons: p110β vs. p110δ (β/δ); Brown Half-filled rectangles: within full-length Class IA adaptors p85α/p85β/p55γ; Magenta inverted blank triangles: p85α vs. p85β; Black filled diamonds: full length p101 vs. full length p84; Asterisks: domains in p101 vs. domains in p84.

The first crystal structures of PI3K kinases, both with [64] or without [51] inhibitors (PDB: 1E7U, 1E8W, 1E8X, 1E8Y, 1E90; 2.2Å), were resolved by Walker et al. These studies focused on porcine p110γ with the N-terminal ABD deleted. as the full-length p110γ monomer was unstable when expressed without adaptors in insect cells. Shortly thereafter, Pacold et al. determined the crystal structure of human p110γ in complex with RAS (PDB: 1HE8; 3.0Å) [3]. Possibly owing to technical difficulties in crystalizing p110γ, the 3D structures of heterodimeric PI3Kγ (p110γ/p101) were only recently visualized using cryo-EM (PDB: 7MEZ; 2.9Å) [62]. Following these initial studies, the crystal structures of human PI3Kα (p110α/p85α-niSH2; PDB: 2RD0; 3.1Å) and murine PI3Kβ (p110β/p85β-icSH2; PDB: 2Y3A; 3.3Å) have been resolved [57,58]. In these studies, only SH2 domains of adaptors were included to facilitate protein crystallization, as their N-terminal regions of adaptors including SH3 and BH domains are challenging to crystalize. Similar to the case of p110γ crystallization, 3D structures of murine p110δ (PDB: 2WXF; 1.9Å) were acquired by deleting the ABD to stabilize p110δ [65]. In this review, we focus on human PI3K kinases despite high sequence homology of PI3K proteins across species [18,19,20,21,22,23,24,25,26,27]. Using AlphaFold [66], we predicted 3D structures of human Class I kinases based upon their amino acid sequences and analyzed them using ChimeraX-1.8 Matchmaker. While Class I kinases are globally similar (Figure 6A), their 3D structures showed significant differences, with a root-mean-square-deviation (RMSD) exceeding 20Å (Figure 7A, full-length and IA/IB). However, p110β and p110δ were more structurally similar with an RMSD of less than 5Å (Figure 7A, β/δ). A summary of PI3K protein domains and their functions is provided in Table 1.

#### 2.3.1. Structural Details and Potential Functions of ABD

**Figure 8:**
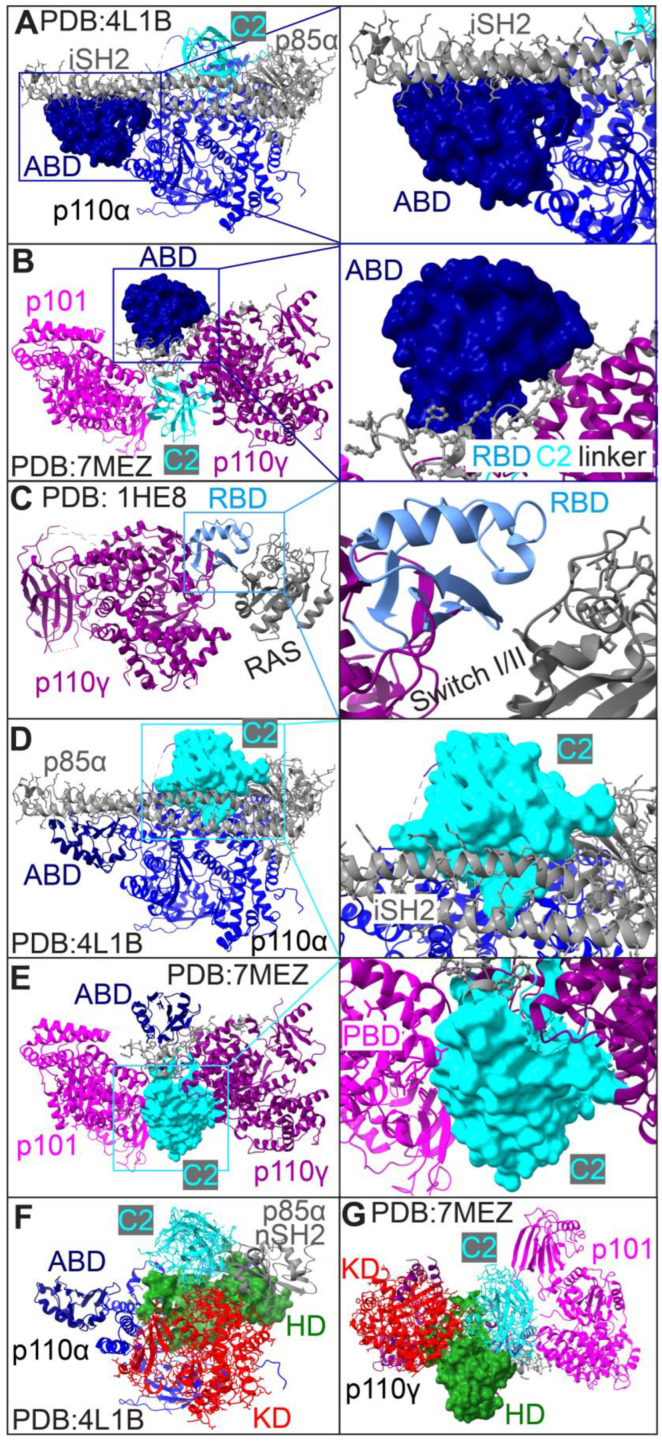
Intra- and inter-subunit interactions in PI3K protein complexes. 3D structures of PI3K protein complexes were retrieved from the PDB database and analyzed using ChimeraX-1.8. Full structures are displayed in the left panel, while the right panel highlights enlarged structures with detailed interface interactions. The interactions include: (**A**) Between p110α’s ABD and p85α’s iSH2; (**B**) Between p110γ’s ABD and RBD C2 linker; (**C**) Between p110γ’s RBD and RAS; (**D**) Between p110α’s C2 and p85α’s iSH2; (**E**) Between p110γ’s C2 and p101′s PBD; (**F**) Between p110α’s HD and p110α’s C2/KD/p85α’s nSH2; (**G**) Between p110γ’s HD and p110γ’s C2/KD/p101. PDB access numbers for representative structures are provided.

As shown in Figure 2 and Figure 4A, the ABDs of Class IA kinases consist of the same number of amino acids, while the ABD of Class IB is slightly longer (α: 16–105, β: 26–115, δ: 16–105, and γ: 34–141). The secondary structures between IA and IB ABDs also differ slightly, with IA ABDs containing five β-sheets and one α-helix, compared to five β-sheets and three α-helices in IB. Despite these differences, IA ABDs share 71% sequence homology within the subclass, with 81–87% similarity among individual IA kinases. However, IA ABDs show only 38% sequence homology with the IB ABD (Figure 5A). When full-length kinases are aligned using ChimeraX-1.8 Matchmaker, the IB ABD (Figure 5A, purple) is orientated differently compared to IA ABDs. Despite this, the 3D shape of the IB ABD is not markedly different from those of IA ABDs, with RMSDs ranging from 2 to 4.5Å between IA ABDs and the IB ABD or among individual IA ABDs (Figure 7A, ABD). These structural differences underpin the functional non-redundancy of IA and IB ABDs. For example, in PI3Kα (PDB: 4L1B) shown in Figure 8A, the ABD of p110α forms multiple inter-subunit interactions with the iSH2 domains in the IA adaptor p85α as reported previously [67]. The globular ABD of human p110α, characterized by an α/β-sandwich topology, is located near one end of the PI3Kα complex, positioned atop the long-rod iSH2-like structure of p85α [56,58]. In addition to the ABD–iSH2 interface, IA ABDs also establish intra-subunit contacts with the KDs via a doubly twisted β-sheet and an α-helix, which involves electrostatic interactions—e.g., R38/R88 of the ABD and E738/D743/D746 of the KD in p110α [54,56,58,68]. In stark contrast, the IB ABD does not bind to the IB adaptors p101/p84 [62], despite its similar protein sequence and 3D shape to IA ABDs. Instead, the IB ABD is proposed to interact with the RBD–C2 linker of p110γ, suggesting a regulatory role in modulating p110γ’s activity (Figure 8B; PDB:7MEZ).

#### 2.3.2. Structural Details and Potential Functions of RBD

RBDs in Class I kinases span different regions, (Figure 2 and Figure 4A) and contain varying numbers of amino acids with different locations: α (187–289), β (194–285), δ (187–277), and γ (217–309). There are 38% of homology between Class IA and IB, 51% of homology within IA, and 68–74% of homology between individual IA RBDs (Figure 5A). Structurally, RBDs exhibit an α/β-fold with antiparallel β-sheets flanked by α-helices (Figure 2). IA RBDs feature four β-sheets separated by three α-helices, while IB RBD’s have five β-sheets and two α-helices. Despite differences in protein sequences and secondary structures, RBDs shared similar 3D shapes (Figure 6A), with RMSDs of 2-5Å between IA and IB RBDs or among individual IA RBDs (Figure 7A, RBD). The RBDs of p110 kinases have shown differential associations with Ras or Rho family GTPases. For instance, p110β’s RBD binds to the Rho GTPase family protein cell division cycle 42 (CDC42), Rac family small GTPase 1 (RAC1), and Ras GTPase RAB5A, member RAS oncogene family [5,69,70]. RBDs of p110α/δ/γ associate with Ras GTPases through different binding interfaces [2,3,4,71,72,73,74]. In p110α, residues K206/T208/K210 in the RBD form salt bridges with E37/S39/R41 in the Switch I region of KRAS proto-oncogene, GTPase (KRAS) [71]. In p110γ, a unique loop in its RBD—absent in p110α/δ—resembles loops found in other Ras-binding proteins such as Raf-1 proto-oncogene, serine/threonine kinase or Ral guanine nucleotide dissociation stimulator [73,74]. This loop interacts with the switch I/II domains in HRAS proto-oncogene, GTPase (HRAS) [3], as illustrated in Figure 8C (PDB: 1HE8). RBDs also exhibit different intra-subunit interactions. For instance, p110α’s RBD is located close to the ATP pocket in the KD [58]; in contrast, p110γ’s RBD does not exhibit this proximity to the KD.

#### 2.3.3. Structural Details and Potential Functions of C2 Domain

C2 domains have been identified in over 100 membrane-interacting proteins primarily known for their calcium-binding function [75]. However, C2 domains in Class I PI3K kinases lack calcium-binding activity and function independently of calcium. These domains are composed of different numbers of amino acids with varying locations: α (330–487), β (327–496), δ (319–476), and γ (357–521). Despite these variations, all C2 domains share a common structural feature: two β-sandwiches, each with four-stranded antiparallel β-sheets (Figure 2 and Figure 4A). Sequence analysis reveals limited homology among C2 domains, with only 36% of similarity between Class IA and IB, 53% of homology within IA, and 53–65% of similarity between individual IA C2 domains (Figure 5A). This diversity makes the C2 domain the most discrete region in p110 kinases. Interestingly, while full-length p110β shares 80% of sequence homology with p110δ, their C2 domains are only 53% similar. Previous research aligns with these findings, showing 36% similarities in IA C2 domains and 27% of identical amino acids between p110α and p110γ [58,63]. 3D structures of human C2 domains, generated by AlphaFold, show diverse loop conformations connecting adjacent β-sheets (Figure 6A and Figure 7, C2), congruent with their varying amino acid compositions (Figure 2. It has been reported [56,58] that the C2 and KD domain in p110α forms a groove accommodating p85α’s iSH2 (Figure 8D; PDB: 4L1B). Notably, residue N345 in p110α’s C2 domain, corresponding to N344 p110β and N334 in p110δ, forms hydrogen bonds with D560/N564 in IA iSH2 domains, which are crucial for kinase stabilization [38]. Additional findings by Rathinaswamy et al. [62] demonstrate that several residues in p110γ’s C2 domain establish close contacts with the PBD domain in p101 (Figure 8E; PDB: 7MEZ), which are critical for stabilizing p110γ. Furthermore, C2 domains are proposed to bind to cellular membrane through positively charged residues enriched in the fully exposed loops (Figure 2 and Figure 6A). IA C2/SH2 domains are suggested to form electrostatic interactions with cellular membranes to activate PI3K signaling [51,53,58,62,65]. This suggestion has been verified in a recent study in which the C2 domain in p110α binds to membrane PIP2 to activate PI3K upon translocation of the PI3Kα complex p110α/p85β onto the endosome membrane [76].

#### 2.3.4. Structural Details and Potential Functions of HD

Although oncogenic mutations are frequently found in the HD domain of p110α [56,77], the precise function of HD domains remains elusive. As shown in Figure 2 and Figure 4A, the HD spans different regions in p110 kinases: α (517–694), β (524–701), δ (497–674), and γ (541–723). Sequence analysis of HD domains shows 53% of homology between Class IA and IB, 63% of homology within IA, and 79–93% of homology between individual IA kinases. Structurally, HD domains are predominantly composed of α-helices (Figure 2). The N-terminal α-helices are arranged perpendicularly (Figure 6A), and the overall structures of Class I HDs are highly conserved with RMSDs of approximately 3Å (Figure 7A, HD). IA HDs form extensive intra-subunit interfaces with the C2 and KD domains. They also interact with nSH2 domains in adaptors, as demonstrated by PI3Kα complexes (Figure 8F; PDB: 4L1B). The HD domain in p110α bridges the C2 and KD domain, constituting the central part of PI3Kα signaling complexes [56,58], These interactions are thought to be crucial for inhibiting p110’s kinase activity by adaptors before activation [67]. Conversely, p110γ’s HD does not interact with p101 (Figure 8G; PDB: 7MEZ), which explains why p101/84 adaptors do not inhibit p110γ’s kinase activity in vitro [26,40]. However, Walker et al. reported that p110γ’s HD interacts with its C2 and KD, which is essential for interdomain packing that stabilizes PI3Kγ complexes [51].

#### 2.3.5. Structural Details and Potential Functions of KD

The KD domains of p110 kinases are the largest domains (Figure 2 and Figure 4A) and are located at the C-termini. They consist of similar number of amino acids: α (765–1051), β (772–1053), δ (745–1027), and γ (797–1080). Among five domains in p110 kinases, KD domains exhibit the highest similarity in protein sequences (Figure 5A, 62–93%) and have the same secondary structures (9–10 α-helices and 8 β-sheets, Figure 2). Consequently, their 3D conformations are highly conserved, with RMSDs of less than 3Å between Class IA and IB or within IA kinases (Figure 7A, KD). Notably, p110α’s KD is less homologous to p110β or p110δ with RMSDs of approximately 4.5Å, whereas p110β’s KD shares high homology with p110δ with an RMSD of around 1.5Å. Earlier studies also report that RMSDs between IA KDs and the IB KD range from 1.6 to 3.2Å [56,58]. Structurally. Class I KD domains share a common 3D arrangement typically found in lipid kinases [78]. This layout comprises a small N-terminal lobe (N-lobe), a large C-terminal lobe (C-Lobe), and a hinge region (cleft) connecting the two lobes. The phosphate-binding loops (P-loops) are primarily located in the N-lobe (Figure 2): α (765–780), β (772–787), δ (745–760), and γ (797–812), overlapping with the inhibitor pocket where PI3K inhibitors bind [79]. The C-lobe contains ATP-binding sites: α (798–807/833–841), β (801–810/836–844), δ (774–783/809–817), and γ (829–838/864–872). The catalytic loop and the activation loop, collectively referred to as the CA-loop (Figure 2), are also located in the C-lobe: α (912–920/931–957), β (916–924/935–961), δ (890–898/909–935), and γ (943–951/962–988). The P-loops and ATP binding sites form an ATP pocket in the cleft, accommodating ATP and PIP2 (Figure 7A, KD). Class I ATP binding sites are highly conserved, with 79–84% of homology (Figure 5A) and RMSDs of less than 1Å (Figure 7A, ATP binding). In contrast, the C-lobe CA-loop shows greater structural variation, with RMSDs ranging from 5–11Å (Figure 7A, CA-loop), despite 93–100% sequence homology (Figure 5A). These subtle differences among Class I KD domains make developing isoform-selective inhibitors challenging, as many PI3K inhibitors target these regions. Maheshwari et al. demonstrated that residue K776 in the p110α’s P-loop recognizes lipid and ATP, regulating phosphate transfer between PIP2 and ATP. Additionally, residue H917 in the catalytic loop and H936 in the activation loop are essential for protein kinase activity [80]. p110α’s K776 corresponds to p110β’s M783, p110δ’s M756, and p110γ’s K808, while p110α’s H917–H936 corresponds to p110β’s H721–H941, p110δ’s H895–H914, and p110γ’s H948–H967. These residues are highly conserved among Class I p110 kinases (Figure 2). Furthermore, Huang and Miled et al. reported that p110α’s ABD, together with p85α’s nSH2/iSH2, brings p110α’s RBD close to the ATP pocket [56,58], a configuration not observed in p110γ/p101 complexes [51]. Additionally, p110α’s C2 and KD, along with positively charged residues in p85α’s iSH2 domain, form electrostatic contacts with negatively charged phospholipids on the membrane, facilitating kinase activation [58]. In summary, Class I KDs are highly conserved, multi-functional domains crucial for kinase activity, lipid recognition, and protein stabilization.

### 2.4. 3D Structures of PI3K Adaptors

Structural studies on PI3K proteins initially focused on individual domains of adaptor proteins. The first structure was the SH2 domain in p85α (nSH2, PDB: 2PNB), determined using NMR in 1992 [43]. While not entirely accurate (Figure 3), this NMR structure featured with two α-helices flanking three β-sheets connected by loops. Subsequent studies used X-ray crystallography to resolve more individual domains of PI3K adaptors, including SH3 (PDB: 1PHT; 2.0Å) [47], BH (PDB: 1PBW; 2.0Å) [55], nSH2 (PDB: 7RNS; 2.0Å) [48], iSH2 (PDB: 2V1Y; 2.4Å) [56], and cSH2 (PDB: 1H9O; 1.8Å) [81]. Despite these advances, no full-length adaptor proteins have been structurally resolved using NMR or X-ray crystallography, likely due to their low stability in solution.

#### 2.4.1. N-Terminal Domains of Class IA Adaptors

While full-length p85α and p85β share 79% homology in their amino acid sequences (Figure 3 and Figure 5B), the homology of their N-terminal domains is significantly lower (56–63%), with the exception for the SH3 domain, which exhibits 80% of homology (Figure 5B). This lower N-terminal homology may may contribute to their differing roles in regulating PI3K signaling. Based on structures revealed through NMR [44,45,47], the SH3 domain adopts a compact β-barrel structure. The SH3 domains of p85α and p85β are nearly identical, with an RMSD of less than 1Å (Figure 7B, SH3), which is consistent with their high sequence homology. The SH3 domain binds to a 21-residue peptide motif in the GTPase dynamin, the motif RKLPPRPSK frequently found in ligands, which adopts a polyproline-II helix conformation, as well as the proline-rich motif in the CBL proto-oncogene [44,47,82]. The P1/P2 domains are enriched with proline residues (Figure 3). While no experimental structures are currently available, AlphaFold predictions suggest that the P1/P2 domains consist of a single α-helix or β-sheet. Interestingly, these predicted structures do not align well between p85α and p85β (Figure 6B), suggesting notable structural differences. P1/P2 domains serve as binding sites for other SH3-containing non-RTKs, connecting PI3K signaling with other signaling pathways [82]. The BH domain comprises seven large α-helices and three small α-helices. It serves as a binding site for GTPase-activating proteins [83] or the Rho GTPase family proteins CDC42 and RAC1 [5,55]. Notably, the BH3 domain in p85α differs significantly from that of p85β with an RMSD of 8Å (Figure 7B, BH).

#### 2.4.2. C-Terminal Domains of Class IA Adaptors

The three SH2 domains—nSH2, iSH2, and c-SH2—located at the C-terminus of p85α and p85β are also present in the shorter isoforms p55α, p50α, and p55γ (Figure 4B). These SH2 domains harbor 91% to 98% of homology (Figure 3 and Figure 5B). Structurally, the 3D conformations of nSH2 and cSH2 were nearly identical among Class IA adapters, with RMSDs of less than 1Å (Figure 7B, nSH2/cSH2). However, the iSH2 domains are less conserved, with RMSDs of approximately 7Å (Figure 7B, iSH2). Upon activation, the nSH2 and cSH2 domains recognize and bind to phosphotyrosine motifs (pYXXM), which are frequently found in RTKs such as epidermal growth factor receptor (EGFR), hepatocyte growth factor receptor, platelet-derived growth factor receptor, and insulin receptors. This binding facilitates the recruitment of PI3K complexes to the plasma membrane [43,46,48,49], an essential step for lipid modification reactions catalyzed by PI3K kinases. Supporting this concept, it has been observed that N-terminal myristoylation or C-terminal isoprenylation of p110 kinase proteins can lead to adaptor-independent membrane translocation and constitutive activation of PI3K [42]. These observations suggest that PI3K adaptors play dual roles in regulating PI3K signaling: (1) Adaptors lock kinases in an activity restrictive state in the cytosol and (2) Upon translocation to the membrane, adaptors change the conformation of PI3K complexes to release unlock and activate kinases. Synthetic phosphopeptides, such as the bisphosphopeptide DDCPYMPMSPGAGAGAGAGAGNGDPYMPMSPKS derived from RTKs, have been used in cell-free kinase activity assays as activators [35,38]. These peptides can bind to nSH2 or cSH2, inducing conformational changes in adaptors that lead to kinase activation. The iSH2 domains in Class IA adaptors share 91% of homology (Figure 3, Figure 4B and Figure 5B) and have two long α-helices flanked by two short α-helices, forming a rod-like structure (Figure 6B and Figure 7B). It is proposed that the iSH2 domain acts as a scaffold for kinase docking [22,36,38,41,52]. However, it appears to have no direct effect on the kinase activity, as phosphopeptides fail to activate kinases when only iSH2 domain is present [84]. This conclusion has been challenged by the finding that viral nonstructural protein 1 binds to the iSH2 domain, disrupting inhibitory contacts between nSH2 and kinases and thereby activating PI3K [85]. Thus, while the iSH2 domain alone may not directly inactivate the kinase, it is essential for the stability of signaling complexes. Class IA adaptors regulate p110 kinases via diverse interactions. For example, p85α’s nSH2 and iSH2 domains interact the HD and KD domains of p110α to mask its activity [38,50]. In contrast, all three SH2 domains—nSH2, iSH2, and cSH2 in p85α/p85β—interact with p110β or p110δ, regulating their activities [57].

#### 2.4.3. Domains in Class IB Adaptors

The Vogt group identified two functional domains within truncated Class IB adaptor p101: the PBD at the N-terminus and GBD at the C-terminus [86]. These domains span the residue 25–175 and 650–850, respectively (Figure 3). The secondary structures of the PBD domain feature a helical solenoid and an α/β barrel structure, whereas the GBD domain comprises an α/β sandwich. Class IB PI3K signaling is predominantly driven by Gβγ signaling [24,26,37,62]. Advancements in cryo-EM and hydrogen-deuterium exchange mass spectrometry (HDX-MS) have provided insights into the 3D structures of PI3Kγ complexes. Rathinaswamy et al. utilized a nanobody to stabilize the GBD domain in p101, enabling the visualization of the PI3Kγ (p110γ/p101) complex via cryoEM [62]. Their studies confirmed the previously identified interaction between p101’s PBD and the C2 domain in p110γ [86]. Additionally, they discovered a new interface between the GBD domain in p101 and the C2 domain in p110γ. Differing from the Class IA adaptors, which significantly affect the stability and basal activity of their associated kinases, Class IB adaptors p101 and p84 do not influence the stability or basal activity of p110γ [62]. Interestingly, while the N-terminus of p110γ shares similar protein sequences and predicted structural similarity with the ABDs of IA kinases (Figure 2 and Figure 7A), it does not form inter-subunit interactions with p101 or p84. Instead, the N-terminal domain of p110γ interacts internally with the linker region between the RBD and C2 domains (Figure 8B). Further research is needed to fully characterize the structural dynamics of PI3Kγ complexes and to deepen our understanding of the regulatory mechanism governing this PI3K signaling pathway.

## 3. Structural Basis of Class I PI3K Signaling in Oncogenic Transformation

### 3.1. Functional Divergence of PI3K Kinases

Class I PI3K kinases, while structurally homologous, exhibit significant functional divergence, which is thought to originate from their differential expression patterns or mutation landscapes in various cancers. For example, p110α and p110β are broadly expressed in many tissues and are crucial for oncogenic transformation, particularly in solid tumors [87]. In stark contrast, p110δ and p110γ are primarily expressed in immune cells and contribute to diseases such as rheumatoid arthritis [88], asthma [89], and hematopoietic malignancies [90,91]. Although p110α and p110β are often co-expressed in tissues, their functions are neither redundant nor interchangeable [92,93,94,95,96]. For instance, p110α is the primary PI3K kinase that activates PI3K signaling downstream of insulin receptors [92,94], which is why inhibitors targeting p110α induce hyperglycemia in patients [97]. On the other hand, p110β is dispensable for insulin signaling but is crucial for platelet production and activity [98], and inactivation of this isoform can increase bleeding risk [90,91]. Additionally, p110β has unique roles in regulating DNA replication [99], male fertility [100], and oncogenic transformation [91,96]. The functional divergence of the p110 isoforms has been summarized in other works [101] and will not be discussed in detail here. One of the most significant functions of PI3K signaling is its role in oncogenic transformation due to its essential contribution to cell survival. In fact, studies have shown that homozygous ablation of *Pik3ca* or *Pik3cb* gene in mice (p110α^−/−^ or p110β^−/−^) is embryonic lethal, causing death occurring between E9.5 and E10.5 [90,102]. The complex role of PI3K signaling in oncogenic transformation is discussed further below.

### 3.2. Oncogenic Potential of Mutant PI3K Proteins

#### 3.2.1. Oncogenic Mutations in PI3K Kinases

**Figure 9:**
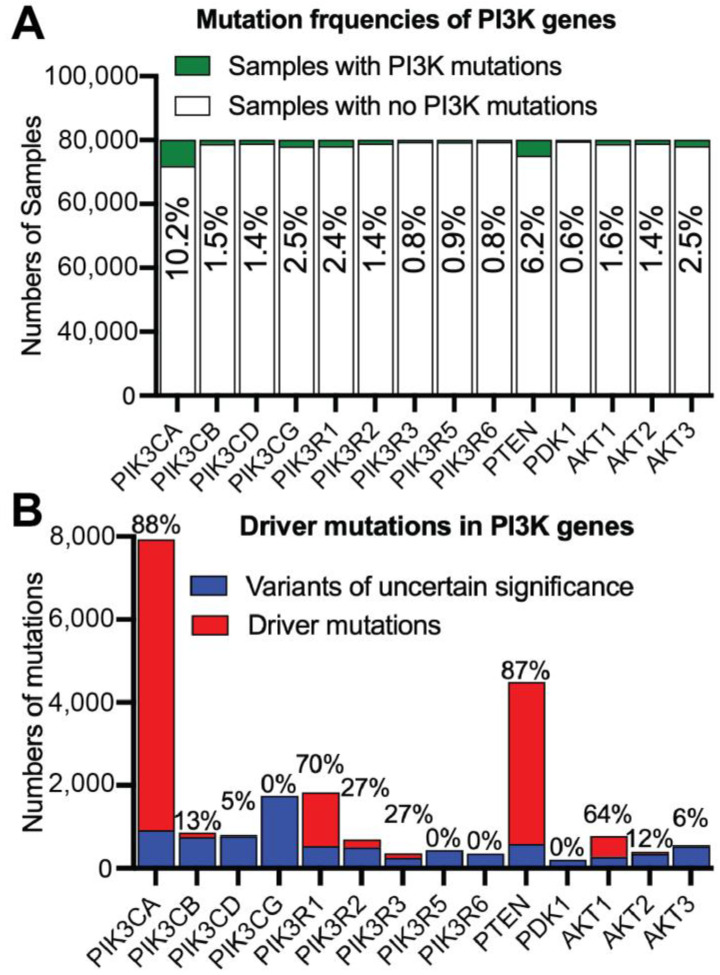
Mutations in PI3K genes in cancer. Data were retrieved from cBioportal, encompassing 800,085 tumor samples across 149 tumor types from 224 studies. (**A**) Mutation frequencies of PI3K genes. The figure shows the number of samples with or without mutations in PI3K genes as well as mutation frequencies. Mutation frequencies were calculated by dividing the number of samples with PI3K mutations with the total number of samples. (**B**) Driver mutations in PI3K genes. Shown are numbers of mutations either recognized as driver mutations or variants of uncertain significance. Frequencies of driver mutations, which were obtained by dividing the number of driver mutations by the total number of mutations, are also shown.

**Figure 10:**
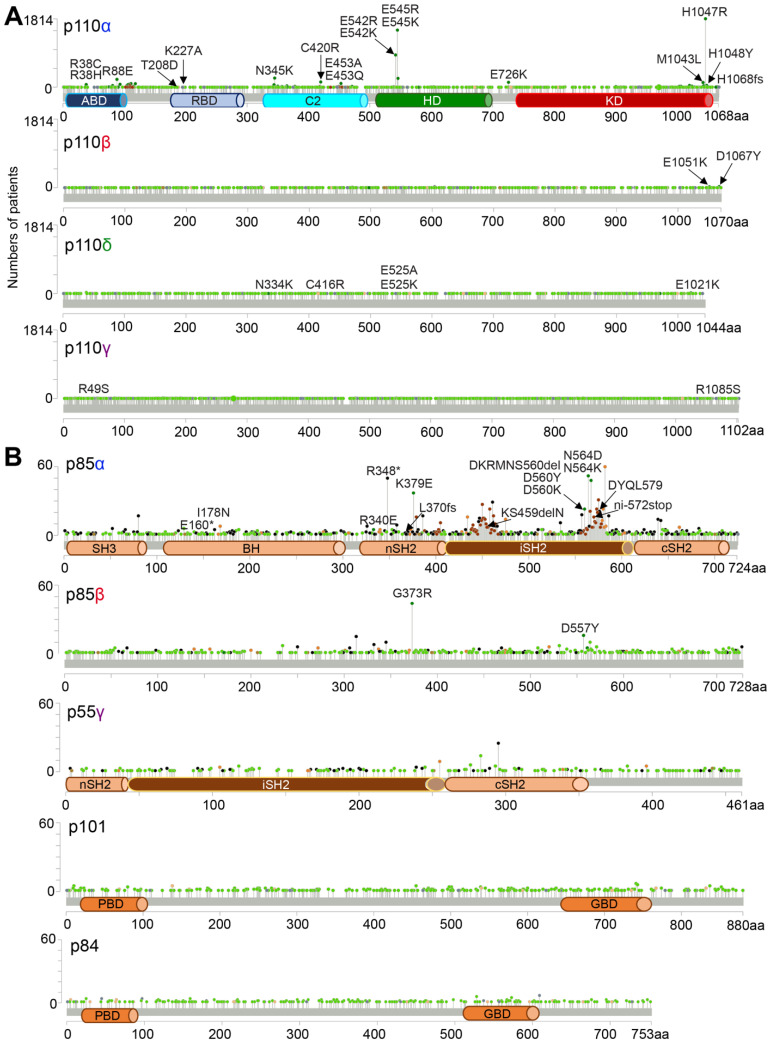
Hotspot mutations in PI3K proteins. Data were retrieved from cBioportal, Mutation plots for PI3K kinases (**A**) or adaptors (**B**) were extracted from cBioportal and replotted. Shown are hotspot mutations in functional domains of p110α, p110β, p110δ, p110γ, p85α, or p85β. *: stop-codon. Dark colors: driver mutations; Light colors: variants of uncertain significance; Dark or light green dots: missense mutations; Black or grey dots: truncations; Dark or light brown dots: in-frame mutations; Dark or light orange dots: splice mutations; Dark or light purple dots: fusion mutations.

PI3K signaling is one of the most frequently mutated oncogenic signaling pathways in cancer. Analysis of approximately 80,000 tumor samples from 224 studies (cBioportal) reveals that 10.2% and 6.2% samples bear mutations in PIK3CA and PTEN (Figure 9A), making these the most frequently mutated PI3K genes. Correspondingly, 88% and 87% of mutations in these genes are classified as driver mutations (Figure 9B), although PIK3R1 and AKT1 also exhibit high frequencies of driver mutations. Oncogenic mutations in p110α, such as p110α^H1047R^ and p110α^E545K^ (Figure 10A), make it an attractive drug target [103,104]. Previous summaries have outlined p110α somatic mutations in cancer and the development of inhibitors targeting both wild-type and mutant p110α [105]. A novel allosteric p110α inhibitor, STX-478, has been discovered, showing preferential binding to p110α^H1047R^ over wild-type p110α or p110α^E545K^ [106]. This underscores the oncogenic role of p110α mutants and the need for selective targeting to mutated p110α proteins to enhance cancer treatment [107]. In contrast, mutations in other Class I p110 kinases are less frequent, averaging less than 5%, with a range of 0% to 10% (Figure 9A). Oncogenic hotspot mutations in p110β are rare (Figure 10A), but include mutations such as p110β^D1067V^, p110β^D1067Y^, and p110β^E1051K^ [108,109,110]. Mutations in p110δ including p110δ^E334K^, p110δ^C416R^, p110δ^E525K^, p110δ^E525A^, and p110δE^1021K^, are also rare (Figure 10A). These mutations typically induce a heterozygous gain of function, leading to activated PI3Kδ syndrome 1 [111], and predisposes patients to B-cell lymphoma [112]. Similarly, heterozygous germline mutations like p110γ^R49S^ and p110γ^N1085S^ (Figure 10A) have been reported in an immunodeficient patient [113], though these mutations have not been linked to haemopoietic malignancy. Interestingly, p110γ has been proposed as a tumor suppressor in colorectal cancer, with genetic ablation in mice leading to spontaneous colorectal adenocarcinoma [114], although this role has not been confirmed in human colorectal cancers.

#### 3.2.2. Oncogenic Mutations in PI3K Adaptors

Among PI3K adaptors, p85α exhibits the most frequent genetic alterations, with a mutation frequency of 2.4% (Figure 9A) and a driver mutation frequency of 70% (Figure 9B), higher than that of other adaptors. These mutations, especially in the nSH2 and/or iSH2 domains, are notably common in endometrial cancer, where approximately 30% of cases feature mutations in PIK3R1 gene [115]. Specific mutations, including deletions or single-residue changes, can activate PI3K signaling, contributing oncogenesis. For instance, deletions or truncations of the C-terminal cSH2 domain (Figure 10B) have been shown to enhance the transformation potential of nSH2 mutants such as p85α^K379E^ and p85α^R340E^ [116]. Mutations in exon 11 of the PIK3R1 gene can result in a shortened iSH2 domain (e.g., deletions of residue 434–475), leading to the release of PI3Kδ inhibition [117]. This causes type 2-activated PI3K syndrome, associated with an increased risk of lymphoma, similar to the mutations in PI3Kδ that cause activated PI3Kδ syndrome 1. Additionally, hemizygous deletion of the PIK3R1 gene, commonly observed in breast cancer, has been shown to transform human mammary epithelial cells [118]. The aforementioned mutations suggest that PIK3R1 acts as a tumor suppressor by inhibiting oncogenic p110 kinases. Defective p85α and its splicing isoforms often result in constitutive activation of PI3K signaling, driving oncogenesis. However, mutations in the N-terminal domains of p85α, such as SH3 and BH, have been reported to exhibit p110-independent activities, activating other survival signaling pathways that may promote oncogenic transformation [119,120].

#### 3.2.3. Molecular Basis of Oncogenic PI3K Kinase Mutants

Hotspot mutations in p110α occur across different domains (Figure 10A), each leading to distinct mechanisms of oncogenic activation. Mutations such as p110α^R38C^, p110α^R38H^, and p110α^R88E^ in the ABD domain alter the interface between the ABD and KD domains [58,121], suggesting that ABP plays an important role in the constitutive activation of p110α. In the C2 domain, two hotspot mutations—p110α^N345K^ and p110α^E453Q^—disrupt the interaction between the C2 and iSH2 domains, leading to the constitutive activation of PI3K signaling [58]. Further evidence shows that mutations like p110α^C420R^, p110α^E453A^, and p110α^E453K^ change intra- and inter-subunit interactions, activating cellular transformation [122,123]. Mutations in the HD domain, including p110α^E542R^, p110α^E542K^, p110α^E545R^ and p110α^E545K^, are among the most active and oncogenic. These mutations disrupt the contact between the HD domain and the nSH2 domain in p85α, leading to constitutive activation of p110α [56,77]. However, these mutants still require RAS for transformation, as shown by diminished transformational potential when p110α’s RBD domain, which binds to RAS, is inactivated [124]. The p110α^H1047R^ mutation, located at the activation loop of the KD domain, enhances membrane association by changing the conformation of residue 864–874 and directly binding to charged lipids on the membrane [68,125]. This mutation allows for malignant transformation independent of RAS, contrasting with the mechanisms of p110α^E542K^ and p110α^E545K^ [124]. Interestingly, p110γ has an arginine residue at 1076, similar to p110α^H1047R^. However, unlike p110α^H1047R^, which is situated on a more accessible loop, this residue in p110γ is buried in a helix. This difference underscores the importance of membrane association in the constitutive activation of p110α^H1047R^ [68]. Vasan et al. reported that p110α^E726K/H1047R^, p110α^E545K/E726K^, p110α^E545K/M1043L^, or p110α^E453Q/H1047R^ exhibited significantly strong oncogenic potential. These double mutants sustain PI3K activation independent of growth factors, enhance the association with PIP2 on the membrane, and increase transformation in MCF10A and NIH3T3 cells [126]. Liu et al. used cryo-EM to reveal the structures of p110α^E542K^, p110α^E545K^, and p110α^H1047R^, confirming the key interactions that contribute to their oncogenic activity [127]. Recently, a novel oncogenic mutation, p110α^H1048Y^, was identified in the KD domain of p110α in breast cancer patients [128]. Additionally, a frameshift mutation, p110α^N1068fs^, which elongates the p110α protein with additional arginine and lysine residues at the C-terminus, has been discovered [129]. This longer p110α mutant protein, like p110α^H1047R^, constitutively activates PI3K and transforms human mammary epithelia cells in a p85-dependent and RAS-independent manner.

#### 3.2.4. Molecular Basis of Oncogenic Mutants of PI3K Adaptors

Active mutations in the PIK3R1 gene are commonly clustered in the nSH2 and iSH2 domains [115,130,131], due to their essential role in allosterically regulating the inter-subunit interactions (e.g., nSH2 and C2 or iSH2 and ABD/C2) within PI3K signaling complexes [56,58,68]. These mutations frequently result in the release of p110 kinase inhibition by adaptors, leading to constitutive activation of p110 kinases and cellular transformation. For example, Hofmann and Jucker studied mutations in the nSH2 and cSH2 domains of p85α and found that while the nSH2 mutants p85α^K379E^ and p85α^R340E^ activated p110α, their oncogenic transformation potential in chicken embryo fibroblasts increased substantially when the cSH2 was deleted. This suggest that the combination of these mutations fully abolish the inhibitory interactions between p85α and p110α [116]. Interestingly, while lysine residues in p110α^E542K^ or p110α^E545K^ create an electrostatic clash with K379 in wild-type p85α, which displaces p110α’s HD from p85α’s nSH2, the p85α^K379E^ mutation reestablishes interactions with these p110α mutants, counteracting their oncogenic transformation [56,132]. The oncogenic role of cSH2 truncations was confirmed by Jimenez et. al., who found that the p85α mutant p65α, which lacks the cSH2 domain, transforms NIH3T3 cells together with p110α [133]. Hao et al. also observed that p110α^E542K^ or p110α^E545K^ in colon cancers depend on nuclear translocation and epigenetic regulation of p85β, but not p85α, to exert their oncogenicity. This indicates a complex regulation of PI3K mutations in cancer [134]. Mutations in the iSH2 domain (Figure 10B), such as p85α^D560K^, p85α^D560Y^, p85α^N564D^, p85α^N564K^, p85α^N564D/D569Y^, p85α^DYQL579^, or p85α^ni−572stop^, disrupt the ABD/iSH2 interface, similar to the effect seen in the ABD mutant p110α^N345K^ [53,58,121,135]. This disruption leads to the constitutive activation of p110 kinases and neoplastic transformation. Other iSH2 mutants (Figure 10B), such as p85α^KS459delN^ and p85α^DKRMNS560del^, interfere with the interaction with the C2 domain in p110 kinases and contribute to the transformation of chicken embryo fibroblasts with a potential equivalent to the p110α^H1047R^ [132]. These mutations primarily impact p110α, as cells bearing these mutations are sensitive to p110α’s inhibitors but not inhibitors of p110β or p110δ. While most p85α mutations affect p110 kinases, recent studies have identified p110-independent functions of p85α. For example, mutations in the BH3 and SH3 domains, such as p85αE160* or p85αI178N (Figure 10B), inhibit the formation of p85α dimers, impacting its p110-independent association with PTEN, RAB5, CDC42, dynamin and other p85α-binding proteins [119,120,136]. Cheung et al. found that two recurrent mutations, p85αR348* or p85αL370fs (Figure 10B), result in the nuclear translocation of mutant p85α proteins, subsequently activating the c-Jun N-terminal kinase pathway in the nucleus [137]. Given that liver cells contain 30% more p85α and mammary epithelial cells contain 20% more p85β than p110 kinases [138], it is not surprising that these free adaptors can act independently of p110 kinases once mutated.

### 3.3. Oncogenic Potential of Wild-Type PI3K Proteins

#### 3.3.1. Wild-Type PI3K Proteins Cooperate with Other Oncogenes in Oncogenic Transformation

The earliest evidence demonstrating the involvement of wild-type, nonmutated PI3K proteins in oncogenic transformation is their binding to the oncogene polyoma virus middle T antigen. This interaction induces PI3K activation and transformation in NIH3T3 cells [17]. In this study, mutants of middle T antigen that fail to interact with PI3K proteins and activate PI3K signaling are unable to transform NIH3T3 cells. Similarly, in hematopoietic 32D cells, the introduction of the oncogene BCR-ABL activates RAS and PI3K, both of which are required for the transformation of 32D cells into cancerous cells. This is evidenced by the cytokine-independent growth of these cells, further verifying the essential role of PI3K signaling in oncogenic transformation [139]. Oncogenic RAS, which activates PI3K [4], differentially regulate the activity of p110 kinases, and these PI3K kinases play divergent roles in RAS-activated oncogenic transformation. For example, oncogenic p110α mutations, such as p110α^T208D^ and/or p110α^K227A^ in the RBD domain (Figure 10A), disrupt the interaction between RAS and p110α. This disruption inactivates PI3K signaling in mouse embryo fibroblasts (MEFs) and prevents the transformation of MEFs expressing oncogenic EGFR^L858R^ (which signals through both RAS and PI3K) or HRAS^G12V^, but not polyoma virus middle T antigen. Furthermore, these mutations inhibit the growth of KRAS-driven lung cancer in mice [140]. Wu et al. found that p110α/RAC1, but not p110β, is vital for KRAS^G12D^-driven pancreatic cancers [141]. Together, these studies have established p110α as the key player downstream of oncogenic RAS. However, Kang et al. reported that wild-type p110β, p110δ, and p110γ, but not p110α, can transform chicken embryo fibroblasts independent of myristylation. Transformation by p110β or p110γ still requires RAS-binding [142]. Although p110γ is mainly activated by the GPCR G_β/γ_, full activation of PI3K signaling requires both the association of G_β/γ_ with the adaptor p101 and the binding of RAS to the RBD of p110γ [143].

#### 3.3.2. Wild-Type p110β Outcompetes Other p110 Kinases in Oncogenic Transformation

Among the four p110 kinases, p110β stands out for its exceptional oncogenic potential, rivaling even hotspot mutations in p110α discussed earlier [96,144,145,146]. This distinctiveness likely stems from the functional and structural differences between p110β and other p110 kinases. Unlike p110α, p110δ, or p110γ, which are activated by either RTKs or G_βγ_, p110β can be activated by both [95,147,148,149]. While RTK-activated p110 kinases exhibit varied activities, G_βγ_-activated p110β is functionally redundant to G_βγ_-activated p110γ in stimulating neutrophils [150]. Given the differences in RTK- and GPCR-mediated signaling pathways, activated p110 kinases may have distinct effects on cellular transformation. Indeed, both wild-type p110β and p110α mutants (such as p110α^E542K^, p110α^E545K^, or p110α^H1047R^) constitutively activate PI3K and transform NIH3T3 cells, chicken embryo fibroblasts, or human MEFs, whereas wild-type p110α does not [144,146,151,152]. Supporting its strong tumorigenic capacity, deletion of oncogenic p110β inhibits the formation and progression of PTEN-deficient prostate cancer, colon cancer, and brain cancer [96,153,154,155,156,157,158]. Conversely, ablation of other wild-type p110 kinases does not have the same effect on these cancers. Additionally, p110β, in conjunction with p85β, can translocate into the nucleus to regulate DNA replication and repair via an as-yet undefined mechanism [99,159,160]. A nuclear localization signal “KVKTKKSTK” has been identified exclusively in the C2 domain of p110β (Figure 2). While it is hypothesized that the nuclear PI3Kβ complex contributes to oncogenic transformation through DNA replication and repair, current evidence does not support this hypothesis.

#### 3.3.3. Structural Basis of Oncogenic p110β

By comparing the structures of p110α and p110β, Zhang et al. identified differences in intra- and inter-subunit interactions that account for the heightened oncogenic potential of p110β [57]. The kinase activity of p110 proteins is typically restricted by inter-subunit inhibitory interfaces, referred to as “brakes”, which include: [1] The interfaces between the nSH2 domain and the C2/HD/KD domains; [2] The interactions between the cSH2 domain and the C-lobe in the KD domains; and [3] The contacts between the iSH2 domain and the C2 and KD domains. While these regulatory brakes affect all PI3K complexes, Dbouk et al. identified additional interactions between p110β and its adaptors that render these brakes ineffective and increase its oncogenic potential. Firstly, the residue D445 in the iSH2 domain of p85β interacts with K942 and F943 located on the activation loop of p110β, enhancing the ATP accessibility of the activation loop. Secondly, D551 and N555 in the iSH2 domain of p85β bind to S457 and S458 in the C2 domain of p110β, promoting the membrane association and the constitutive activation of p110β. Lastly, the iSH2-C2 interaction in PI3Kβ complexes exhibits a weaker inhibitory effect on p110β activity compared to similar interactions in other PI3K complexes [146]. Additionally, the C2 domain of p110β contains more basic residues compared to C2 domains of other p110 kinase. This characteristics is analogous to oncogenic mutations in the C2 domain of p110α, which similarly increase basic charge and membrane association [142,144]. For example, the N345K mutation in the C2 domain of p110α induces structural and functional changes that mirror the wild-type p110β, explaining why wild-type p110β shows oncogenic potential equivalent to these p110α mutants [145,146]. Moreover, p110β is unique among p110 isoforms in being activated by both RTKs and G_βγ_. Mutations within the G_βγ_-binding site of p110β (Figure 2, residue 514–537 in the C2/HD linker and the HD domain) disrupt the interaction between G_βγ_ and p110β, weakening p110β’s transformation potential in NIH3T3 cells. This provides further insights into the mechanism by which p110β becomes oncogenic [161].

### 3.4. Targeting Oncogenic p110 Kinases

Since the discovery of PI3K as the key survival signaling pathway in cancer and the recognition of p110 kinases as oncogenes, targeting PI3K signaling has emerged as a compelling therapeutic strategy. Nonetheless, pan-PI3K inhibitors, which non-selectively block all four p110 kinases, have shown limited success due to their inability to account for the differential oncogenic potential of p110 kinases across cancers. This lack of specificity often results in severe side effects and a narrow therapeutic window [79,101]. Of the eighty-four ongoing clinical trials involving PI3K inhibitors for cancer treatment, only three are testing pan-PI3K inhibitors, while the majority focus on isoform-selective inhibitors (Table 2 and Table 3).

The diverse biochemical properties, tissue-specific expression profiles, and distinct roles in tumorigenesis exhibited by the four p110 kinases underscore the need of isoform-specific inhibitors as a more refined therapeutic strategy [89,97,101,106]. Despite this, designing small molecules that target individual p110 kinase isoforms remains a significant challenge. Several p110δ inhibitors, including idelalisib [162], duvelisib [163], and umbralisib [164], were previously approved by the U.S. Food and Drug Administration for treating various types of leukemia or lymphoma, given the dominant role of p110δ in hematopoietic malignancies. However, these drugs have been voluntarily withdrawn recently for certain indications of fatal adverse effects [165,166,167,168]. This clinical setback highlights a key limitation: these small molecules target the ATP-binding domain, a region conserved across p110 kinase isoforms (Figure 7A). Consequently, therapeutic doses of these inhibitors inadvertently affect other PI3K isoforms, causing severe toxicities. To overcome this limitation, a promising approach involves exploiting the structural differences among the p110 kinases, as described in earlier sections. This strategy increases specificity and efficacy while reducing potential off-target effects associated.

Resistance to current cancer treatments, including PI3K inhibitors, is primarily driven by oncogenic mutations and/or the activation of alternative survival signaling pathways (reviewed in [169]). Oncogenic mutations can render PI3K proteins constitutively active and resistant to inhibitors, contributing to cancer progression to advanced stages [170]. Currently, nine ongoing trials are investigating chemical compounds that selectively target mutated PI3K proteins, such as p110α-H1046R, in cancers harboring these mutations (Table 2 and Table 3). Another promising strategy involves exploring the therapeutic potential of isoform-selective PI3K inhibitors in combination therapies. By leveraging synergistic cytotoxic effects, these approaches can reduce the required doses of each agent, thereby minimizing side effects. Notably, drug combination therapies represent the majority of ongoing PI3K clinical trials (Table 2), with sixty-three trials currently testing PI3K inhibitors in combination with other treatments. This trend highlights the potential of combination therapies to improve clinical outcomes. In summary, while the development of isoform-selective PI3K inhibitors remains challenging, the integration of structural insights, targeted approaches for oncogenic mutations, and combination therapies offers a promising path toward overcoming the limitations in current treatments.

## 4. Conclusions and Perspectives

Prior research on Class I PI3K signaling has significantly advanced our understanding of its regulation in both normal and malignant cells. It has also uncovered the molecular mechanisms by which oncogenic PI3K signaling contributes to cancer formation and progression. Notably, structurally homologous p110 kinases have demonstrated divergent functions in cancer, providing a strong rationale for selectively targeting individual p110 kinases or adaptors to enhance cancer treatment. This finding aligns with the concept of precision or personalized oncology, offering avenues for developing more effective PI3K-based therapies.

In this review, we have provided a synopsis of the molecular details of functional domains within PI3K proteins, as revealed through biochemical, structural, and functional analyses. These molecular details shed new light on the dysregulated activity of PI3K in cancer. In summary, three key regulatory levels are crucial for oncogenic activation of PI3K signaling. The first level of regulation involves upstream activators of PI3K signaling. The four p110 kinases respond differentially to upstream activators. p110α and p110δ are generally controlled by RTKs, whereas p110γ is primarily activated by G_βγ_. In contrast, p110β is unique being activated by both RTKs and G_βγ_. Moreover, differential binding capacities of p110 kinases to the upstream activator, the oncogenic RAS, render these kinases distinct potentials in malignant transformation. The second level of regulation is primarily controlled by inter-subunit interactions. Three interfaces between kinases and adaptors (i.e., nSH2-C2/HD/KD, cSH2-KD, and iSH2-C2/KD) serve as brakes to prevent the activation of p110 kinases. Intriguingly, additional inter-subunit interactions observed between p110β and its adaptors render this wild-type, nonmutated protein constitutively active and oncogenic. The third level of regulation comes from genetic mutations in p110α and p85α. Active mutations in p110α or p85α often disrupt restrictive interactions between p110α and p85α, leading to constitutive activation of p110α. These three levels of regulation between PI3K kinases and adaptors, in conjunction with additional regulations from tumor suppressors like PTEN or other oncogenes, establish a tight control over oncogenic transformation in tumorigenesis.

Despite these advances, significant knowledge gaps remain. Many structural insights derive from purified/crystalized proteins, raising questions about whether these findings from purified/crystalized proteins accurately represent the native conformations of PI3K proteins within cells. Furthermore, activated and inactivated PI3K protein complexes have not been visualized at atomic levels, leaving a gap in understanding the dynamic changes of protein conformations within cells and their roles in regulating kinase activities during oncogenic transformation. To address these challenges, further research should leverage cutting-edge techniques like cryo-EM, which enables the visualization of native protein complexes isolated from cells. By capturing the 3D structures of active or inactive PI3K complexes at different stages of cancer evolution, cryo-EM could provide unprecedented insights into the dynamic regulation of PI3K activity. Despite the enthusiasm for targeting PI3K in cancer therapy, clinical success has been limited, largely due to the incomplete understanding of PI3K regulation in cancer. Future molecular and structural investigations will be pivotal in uncovering therapeutic vulnerabilities within PI3K functional domains and motifs. These efforts hold promise for fostering the rational design of effective PI3K-based cancer therapies and overcoming current treatment limitations.

## Figures and Tables

**Table 1:** Synopsis of PI3K protein domains and their functions.

Isoform	Domain /Motif	Location /Length	Secondary Structure	Similarity (RMSD)	Function(s)	PDB /UniProt
p110α	ABD	16–105 90aa	Globular, 1 α-helix/5 β-sheets	1.9Å to β, 1.3Å to δ	Forming ABD–iSH2 interfaces to stabilize complexes; Binding to the KD	4L1B, human P42336
	RBD	187–289 103aa	α/β-fold structures, 3 1 α-helix/4 β-sheets	5.4Å to β, 3.9Å to δ	Binding to the Switch domains in RAS family proteins or other small GTPase	
	C2	330–487 158aa	β-sheets/loops, 8 β-sheets	6.6Å to β, 3.6Å to δ	Forming C2/KD–iSH2 interfaces to stabilize complexes and facilitate membrane translocation	
	HD	517–694 178aa	10 α-helices	2.7Å to β, 2.6Å to δ	Binding C2/KD and nSH2 to mask kinase activity	
	KD	765–1051 287aa	N-lobe/hinge/C-lobe, 9 α-helices/8 β-sheets	4.1Å to β, 3.9Å to δ	Accommodating ATP and PIP2 to catalyze phosphate groups transferring	
	ATP-binding	798–807/833–841, 19aa	KD β3/β4 β-sheets	0.7Å to β, 0.7Å to δ	Binding ATP	
	CA-loop	912–920/931–957, 36aa	KD’s linker between α4 and β7 or β8 and α5	10.6Å to β, 4.7Å to δ	Kinase catalytic center transferring phosphate groups from ATP to PIP2	
p110β	ABD	26–115 90aa	Globular, 1 α-helix/5 β-sheets	1.9Å to α, 1.6Å to δ	Forming ABD–iSH2 interfaces to stabilize complexes; Binding to the KD	2Y3A, murine P42338
	RBD	194–285 102 aa	α/β-fold structures, 3 α-helices/4 β-sheets	5.4Å to α, 2.4Å to δ	Binding to the Switch domains in RAS family proteins or other small GTPase	
	C2	327–496 170aa	β-sheets/loops, 8 β-sheets	6.6Å to α, 11.1Å to δ	Forming C2/KD–iSH2 interfaces to stabilize complexes and facilitate membrane translocation	
	HD	524–701 178aa	10 α-helices	2.7Å to α, 2.8Å to δ	Binding C2/KD and nSH2 to mask kinase activity	
	KD	772–1053 282aa	N-lobe/hinge/C-lobe, 9 α-helices/8 β-sheets	4.1Å to α, 1.5Å to δ	Accommodating ATP and PIP2 to catalyze phosphate groups transferring	
	ATP-binding	801–810/836–844, 19aa	KD β3/β4 β-sheets	0.7Å to α, 0.3Å to δ	Binding ATP	
	CA-loop	916–924/935–961, 36aa	KD’s linker between α4 and β7 or β8 and α5	10.6Å to α, 5.5Å to δ	Kinase catalytic center transferring phosphate groups from ATP to PIP2	
p110δ	ABD	16–105 90aa	Globular, 1 α-helix/5 β-sheets	1.3Å to α, 1.6Å to β	Forming ABD–iSH2 interfaces to stabilize complexes; Binding to the KD	6G6W, human O00329
	RBD	187–277 91aa	α/β-fold structures, 3 α-helices/4 β-sheets	3.9Å to α, 2.4Å to β	Binding to the Switch domains in RAS family proteins or other small GTPase	
	C2	319–476 158aa	β-sheets/loops, 8 β-sheets	3.6Å to α, 11.1Å to β	Forming C2/KD–iSH2 interfaces to stabilize complexes and facilitate membrane translocation	
	HD	497–674 178aa	10 α-helices	2.6Å to α, 2.8Å to β	Binding C2/KD and nSH2 to mask kinase activity	
	KD	745–1027 283aa	N-lobe/hinge/C-lobe, 9 α-helices/8 β-sheets	3.9Å to α, 1.5Å to β	Accommodating ATP and PIP2 to catalyze phosphate groups transferring	
	ATP-binding	774–783/809–817, 19aa	KD β3/β4 β-sheets	0.7Å to α, 0.3Å to β	Binding ATP	
	CA-loop	890–898/909–935, 36aa	KD’s linker between α4 and β7 or β8 and α5	4.7Å to α, 5.5Å to β	Kinase catalytic center transferring phosphate groups from ATP to PIP2	
p110γ	ABD	34–141 108aa	3 α-helices/5 β-sheets	4.5Å to IA	Binding to the RBD–C2 linker; Not binding to the adapter	7MeZ, human P48736
	RBD	217–309 93aa	α/β-fold structures, 2 α-helices/5 β-sheets	4.8Å to IA	Binding to the Switch domains in RAS family proteins or other small GTPases.	
	C2	357–521 165aa	β-sheets/loops, 8 β-sheets	8.8Å to IA	Binding to the PBD domain of adaptor to stabilize complexes	
	HD	541–723 183aa	10 α-helices	2.7Å to IA	Binding to C2/KD to stabilize complexes	
	KD	797–1080 284aa	N-lobe/hinge/C-lobe, 10 α-helices/8 β-sheets	2.9Å to IA	Accommodating ATP and PIP2 to catalyze phosphate groups transferring	
	ATP-binding	829–838/864–872, 19aa	KD β3/β4 β-sheets	0.7Å to IA	Binding ATP	
	CA-loop	943–951/962–988, 36aa	KD’s linker between α4 and β7 or β8 and α5	8.1Å to IA	Kinase catalytic center transferring phosphate groups from ATP to PIP2	
p85α p55α p50α	SH3	3–79, 77aa	1 α-helix/5 β-sheets	0.5Å to β	Binding to ligands	1PHT(SH3);1PBW(BH);7RNS(nSH2);2V1Y(iSH2);1H9O(cSH2), human P27986
	BH	113–301, 189aa	10 α-helices	8.2Å to β	Binding to GTPases	
	nSH2 *	333–428, 96aa	Globular, 2 α-helices/6 β-sheets	0.2Å to β/δ	Binding to RTKs’ pYXXM for membrane translocation; Binding to HD/KD to mask kinase activity	
	iSH2	429–623, 195aa	Rod-like structure, 4 α-helices	7.8Å to β/δ	Forming iSH2-C2/KD interface to stabilize complexes	
	cSH2	624–718, 95aa	Globular, 2 α-helices/6 β-sheets	0.2Å to β/δ	Binding to RTKs’ pYXXM for membrane translocation; Masking kinase activity	
p85β	SH3	4–80, 77aa	1 α-helix/5 β-sheets	0.5Å to α	Binding to ligands	3O5Z(SH3);7RNU(nSH2);3MTT(iSH2), human O00459
	BH	109–295, 187aa	10 α-helices	8.2Å to α	Binding to GTPases	
	nSH2	330–425, 96aa	Globular, 2 α-helices/6 β-sheets	0.2Å to α/δ	Binding to RTKs’ pYXXM for membrane translocation; Binding to HD/KD to mask kinase activity	
	iSH2	426–621, 195aa	Rod-like structure, 4 α-helices	7.8Å to α/δ	Binding to RTKs’ pYXXM for membrane translocation; Forming iSH2-C2/KD interface to mask kinase activity	
	cSH2	622–716, 95aa	Globular, 2 α-helices/6 β-sheets	0.2Å to α/δ	Binding to RTKs’ pYXXM for membrane translocation; Masking kinase activity	
p55δ	SH3	N/A				O92569
	BH	N/A				
	nSH2	65–160, 96aa	Globular, 2 α-helices/6 β-sheets	0.2Å to α/β	Binding to RTKs’ pYXXM for membrane translocation; Binding to HD/KD to mask kinase activity	
	iSH2	161–357, 187aa	Rod-like structure, 4 α-helices	7.8Å to α/β	Binding to RTKs’ pYXXM for membrane translocation; Forming iSH2-C2/KD interface to mask kinase activity	
	cSH2	358–452, 95aa	Globular, 2 α-helices/6 β-sheets	0.2Å to α/β	Binding to RTKs’ pYXXM for membrane translocation; Masking kinase activity	
p101	PBD	25–101, 77aa	Helical solenoid, 4 α-helices	5.7Å to p84	Binding to p110γ’s C2	7MEZ, human Q8WYR1
	GBD	653–753, 101aa	α/β sandwich, 3 α-helices/6 β-sheets	19.2Å to p84	Binding to p110γ’s C2 and Gβγ	
p84	PBD	22–94, 73aa	Helical solenoid, 4 α-helices	5.7Å to p101	Binding to p110γ’s C2	Q5UE93
	GBD	520–613, 94aa	α/β sandwich, 3 α-helices/6 β-sheets	19.2Å to p101	Binding to p110γ’s C2 and Gβγ	

* nSH2 domain in p55α and p50α have different length from that of p85α, which is 64–159/95aa or 1–40/40aa, respectively.

**Table 2:** Ongoing cancer clinical trials using PI3K drugs alone or in combination with other treatments. Summarized here are not-yet recruiting, recruiting, or active/not recruiting clinical trials, which are at phase I to IV.

Isoform	Drug	Combined Interventions *	Types of Cancer **	Trial Number
p110α	Alpelisib	None	Advanced *** BC, NSCLC, and GIC	NCT04591431
	Taselisib		Tumors with mPIK3CA	NCT02465060
	TOS-358		HNSCC, UC, EMC, or HR+/HER2– BC	NCT05683418
p110α	Alpelisib	AI: anastrozole, letrozole, or exemestane; SERD: fulvestrant, or elacestrant;	Advanced BC	NCT05826964
		AI: letrozole	HR+ BC	NCT01791478
		AI: letrozole; CDK4/6i: LEE011	HR+/HER2– advanced BC	NCT01872260
		Chemo: nab-paclitaxel	TNBC with mPIK3CA or mPTEN	NCT04216472
		Chemo: capecitabine	Advanced CRC with mPIK3CA	NCT04753203
		ERA: OP-1250	HR+/HER2– advanced BC	NCT05508906
		FTasei: tipifarnib	HNSCC	NCT04997902
		HER2 AB: trastuzumab or pertuzumab	HER2+ advanced BC with mPIK3CA	NCT04208178
		Ketogenic diet; low carbohydrate diet; SERD: fulvestrant; SGLT2i: canagliflozin	Advanced BC with mPIK3CA	NCT05090358
		MEKi: trametinib	Meningioma	NCT03631953
		MetAP2i: evexomostat; SERD: fulvestrant	HR+/HER2– BC with mPIK3CA	NCT05455619
		PARPi: olaparib (AZD2281)	Advanced solid tumors	NCT05564377
		SERD: fulvestrant	Advanced BC with mPIK3CA	NCT04967248
			HR+/HER2– BC with mPIK3CA	NCT05022342
			HR+/HER2– advanced BC	NCT05501886
			HR+/HER2– BC with mPIK3CA	NCT05631795
		Trop-2 AB: sacituzumab govitecan	HER– advanced BC	NCT05143229
	HS-10352	SERD: fulvestrant	HR+/HER2– BC with mPIK3CA	NCT05504213
	Izorlisib	Chemo: eribulin	HR+/HER2– BC with mPIK3CA	NCT05810870
	Serabelisib	Chemo: nab-paclitaxel; Insulin suppressing diet	Advanced solid tumors with mPIK3CA or mPTEN	NCT05300048
Mutant p110α	Inavolisib	None	Advanced cancers with mPIK3CA	NCT04551521
			Early-stage BC	NCT05332561
Mutant p110α	Inavolisib	SERD: giredestrant	HR+/HER2– early-stage BC	NCT05708235
		Chemo: capecitabine	TNBC	NCT04849364
		HER2 therapy: PHESGO and endocrine therapy	HR+/HER2+ early-stage BC with mPIK3CA	NCT05306041
	RLY-2608	CDK4/6i: ribociclib and palbociclib; SERD: fulvestrant	HR+/HER2– BC with mPIK3CA	NCT05216432
	STX-478		Advanced solid tumors.	NCT05768139
p110α-H1047R	LOXO-783	AI: anastrozole, exemestane, or letrozole; CDK4/6i: abemaciclib; Chemo: paclitaxel; SERD: fulvestrant or imlunestrant	BC and other cancers with PIK3CA-H1047R	NCT05307705
	OKI-219	HER2 AB: trastuzumab; SERD: fulvestrant;	Advanced cancer and advanced BC	NCT06239467
p110β	GSK2636771	None	Tumors with mPTEN	NCT04439149
			Tumors with PTEN loss	NCT04439188
p110β	AZD8186	Chemo: docetaxel	Tumors with mPTEN or mPIK3CB	NCT03218826
	GSK2636771	MEKi: trametinib	Tumors with mPTEN and mBRAF	NCT02465060
	GSK2636771	ICI: pembrolizumab	Advanced melanoma with mPTEN	NCT03131908
p110δ	Linperlisib	None	Large granular TLL	NCT06224257
			iBCL	NCT06343935
			Lymphoma and leukemia	NCT06530550
p110δ	IBI376	CD20 AB: pituximab	iNHL	NCT05073250
	Linperlisib	CD20 AB: obinutuzumab; BCL2i: venetoclax	MCL	NCT06324994
		EZH2i: SHR2554	PTCL	NCT06712173
		HDACi: chidamide	CTCL	NCT06037239
			PTCL	NCT06083701
		ICI: camrelizumab; Chemo: pegaspargase; Steroid: dexamethasone	Advanced NKTL	NCT06376721
	Parsaclisib	HDACi: chidamide	PTCL	NCT05083208
		HDACi: romidepsin	Advanced TCL	NCT04774068
		JAKi: itacitinib or ruxolitinib; BTKi: ibrutinib	B-cell malignancies	NCT04509700
	Roginolisib	BCL2i: venetoclax; CD20 AB: rituximab	CLL	NCT06644183
	Umbralisib	ICI: pembrolizumab	CLL and B-cell NHL	NCT03283137
p110γ	Eganelisib	None	Advanced HNSCC	NCT03795610
p110γ	Eganelisib	ICI: atezolizumab; Chemo: nab-paclitaxel; VEGF AB: bevacizumab	TNBC and RCC	NCT03961698
p110α/δ	Copanlisib	None	Tumors with mPIK3CA or mPTEN	NCT02465060
			Advanced tumors with mPIK3CA	NCT05490771
			Advanced tumors with mPTEN	NCT06400238
			Solid tumors with PTEN loss	NCT06360588
p110α/δ	Copanlisib	Ketogenic diet	FL or EMC with PI3K mutations	NCT04750941
		Chemo: eribulin mesylate	Advanced BC and TNBC	NCT04345913
		ICI: durvalumab; PARPi: olaparib	Advanced solid tumors	NCT03842228
		BTKi: ibrutinib	Advanced PCNSL	NCT03581942
		CD20 AB: obinutuzumab	FL	NCT05387616
		CD20 AB: rituximab	iNHL	NCT02367040
			MZL	NCT03474744
			FL	NCT03789240
		CDK4/6i: abemaciclib; SERD: fulvestrant	Advanced BC	NCT03939897
		ICI: avelumab	Advanced UC	NCT05687721
		ICI: ipilimumab or nivolumab	Tumors with mPIK3CA and mPTEN	NCT04317105
			Advanced solid tumors and lymphoma	NCT03502733
		ICI: nivolumab	iNHL	NCT03884998
		PARPi: niraparib (MK-4827)	Advanced solid tumors	NCT03586661
		SERD: fulvestrant	HR+ EMC or OC	NCT05082025
p110δ/γ	Duvelisib	None	Lymphoma; Leukemia	NCT06530550
	Tenalisib		TNBC	NCT06189209
p110δ/γ	Duvelisib	ATRi: ceralasertib	Advanced solid tumors	NCT05514132
		BCL2i: venetoclax	CLL or SLL	NCT03534323
		CAR-T	NHL and ALL	NCT05044039
		CAR-T: tisagenlecleucel	Advanced DLBCL	NCT04890236
		Chemo: docetaxel	HNSCC	NCT05057247
		CD20 AB: rituximab; Chemo: fludarabine or cyclophosphamide	CLL	NCT02158091
Pan-PI3K	Paxalisib	None	Brain metastases with mutations in PI3K	NCT03994796
	Samotolisib		Pediatric solid tumors and NHLs	NCT03155620
			Solid tumors or NHL with mutations in PI3K pathway	NCT03213678
Pan-PI3K	TL117	Chemo: Paclitaxel	Advanced HNSCC	NCT04843098

* AI: aromatase inhibitor; ATRi: ataxia telangiectasia and Rad3-related protein inhibitor; BCL2i: B-cell leukemia/lymphoma 2 inhibitor; BTKi: Bruton’s tyrosine kinase inhibitor; CAR-T: Chimeric antigen receptor (CAR) T-cell therapy; CD20 AB: antibody against CD20; CDK4/6i: cyclin-dependent kinase 4 and 6 inhibitor; Chemo: chemotherapies; ERA: estrogen receptor antagonist; EZH2i: enhancer of zeste homolog 2 inhibitor; FTasei: farnesyltransferase inhibitor; HDACi: histone deacetylase inhibitor; HER-2 AB: antibody against human epidermal growth factor receptor 2; ICI: immune checkpoint inhibitor; JAKi: Janus kinase inhibitor; MEKi: mitogen-activated protein kinase inhibitor; MetAP2i: methionine aminopeptidase 2 inhibitor; PARPi: poly ADP ribose polymerase inhibitor; SERD: selective estrogen receptor degrader; SGLT2i: Sodium-glucose cotransporter-2 inhibitor; Trop-2 AB: antibody against tumor-associated calcium signal transducer 2; VEGF AB: Vascular endothelial growth factor. ** ALL: acute lymphocytic leukemia; BC: breast cancer; BCL: B-cell lymphoma; CLL: chronic lymphocytic leukemia; CRC: colorectal cancer; CTCL: Cutaneous T-cell lymphoma; DLBCL: diffuse large B-cell lymphoma; EMC: endometrial cancer; FL: follicular lymphoma; GIC: gastro-intestinal cancer; HNSCC: head and neck squamous cell carcinoma; HR+/HER2–: hormone receptor positive and human epidermal growth factor receptor 2 negative; iBCL: indolent B-cell Lymphoma; iNHL: indolent non-Hodgkin lymphoma; MCL: mantle cell lymphoma; mBRAF: mutated B-Raf proto-oncogene, serine/threonine kinase; mPIK3CA: mutated phosphatidylinositol-4,5-bisphosphate 3-kinase catalytic subunit alpha; mPIK3CB: mutated phosphatidylinositol-4,5-bisphosphate 3-kinase catalytic subunit beta; mPTEN: mutated phosphatase and tensin homolog; MZL: marginal zone lymphoma; NHL: non-Hodgkin lymphoma; NKTL: NK/T-cell Lymphoma; NSCLC: non-small cell lung cancer; OC: ovarian cancer; PCNSL: primary central nervous system lymphoma; PTCL: Peripheral T-cell lymphoma; RCC: renal cell carcinoma; SLL: small lymphocytic lymphoma; TCL: T-cell lymphoma; TLL: T-cell lymphocytic lymphoma; TNBC: triple negative breast cancer; UC: urothelial cancer. *** Advanced is herein defined as refractory, recurrent, and/or metastatic.

**Table 3:** PI3K drugs used in ongoing cancer clinical trials.

Drug	Synonyms	Target	Molecular Formula	2D Structure *	PubChem CID
Alpelisib	BYL719, BYL-719, NVP-BYL719, Piqray, Vijoice	p110α	C_19_H_22_F_3_N_5_O_2_S	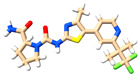	56649450
AZD8186	AZD-8186, AZD 8186	p110β	C_24_H_25_F_2_N_3_O_4_	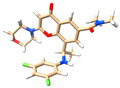	52913813
Copanlisib	BAY 80-6946, BAY-80-6946, Aliqopa	p110α/δ	C_23_H_28_N_8_O_4_	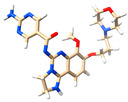	135565596
Duvelisib	IPI-145, IPI145, INK-1197, INK-1147	p110δ/γ	C_22_H_17_ClN_6_O	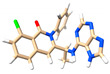	50905713
Eganelisib	IPI-549, IPI549, pi3k-gamma inhibitor IPI-549	p110γ	C_30_H_24_N_8_O_2_	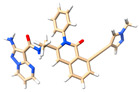	91933883
GSK2636771	GSK-2636771	p110β	C_22_H_22_F_3_N_3_O_3_	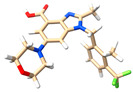	56949517
IBI376	Parsaclisib, Compound 20	p110δ	C_20_H_22_ClFN_6_O_2_	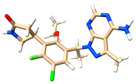	89420683
Inavolisib	GDC0077, GDC-0077, RG6114, RG-6114	Mutant p110α	C_18_H_19_F_2_N_5_O_4_	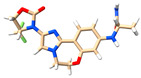	124173720
Izorlisib	MEN1611, CH5132799, CH-5132799, PA-799	p110α	C_15_H_19_N_7_O_3_S	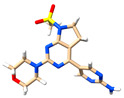	49784945
Linperlisib	Pi3kdelta-IN-2, PI3K(delta)-IN-2, PI3Kd-IN-2, PI3K.DELTA.-IN-2	p110δ	C_28_H_37_FN_6_O_5_S	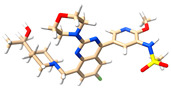	91754520
Parsaclisib	INCB050465, INCB-050465, OS7097575K	p110δ	C_20_H_22_ClFN_6_O_2_	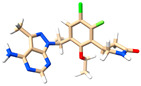	86677874
Paxalisib	GDC-0084, GDC0084, RG-7666, RG 7666	Pan-PI3K	C_18_H_22_N_8_O_2_	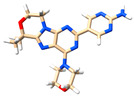	57384863
RLY-2608	RLY2608, EX-A8255, GTPL13065	Mutant p110α	C_29_H_14_ClF_5_N_6_O_2_	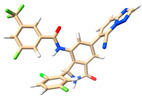	166822065
Roginolisib	IOA-244. IOA244, MSC-2360844	p110δ	C_26_H_27_FN_4_O_5_S	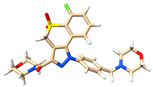	66580799
Samotolisib	LY3023414, LY-3023414, GTPL8918	Pan-PI3K	C_23_H_26_N_4_O_3_	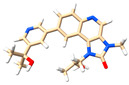	57519748
Serabelisib	MLN1117, MLN-1117, INK1117, INK-1117, TAK-117	p110α	C_19_H_17_N_5_O_3_	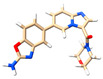	70798655
STX-478	ZWE, STX478, EX-A7997	Mutant p110α	C_16_H_12_F_5_N_5_O_2_	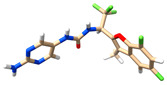	166532451
Taselisib	GDC0032, RG7604, RG-7604	p110α	C_24_H_28_N_8_O_2_	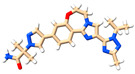	51001932
Tenalisib	RP6530, RP-6530	p110δ/γ	C_23_H_18_FN_5_O_2_	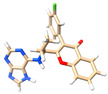	86291103
Umbralisib	TGR-1202, TGR1202, RP5264, RP-5264	p110δ	C_31_H_24_F_3_N_5_O_3_	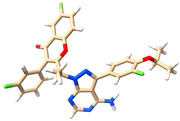	72950888
HS-10352 **	N/A	p110α	N/A	N/A	N/A
LOXO-783 **	N/A	p110α-H1047R	N/A	N/A	N/A
OKI-219 **	N/A	p110α-H1047R	N/A	N/A	N/A
TL117 **	N/A	Pan-PI3K	N/A	N/A	N/A
TOS-358 **	N/A	p110α	N/A	N/A	N/A

* 3D structures of compounds were retrieved from PubChem. Images were exported using ChimeraX-1.8. ** These chemicals were not found in PubChem.

## Data Availability

Results of protein sequences and structures have been presented in this review. Original data or analyses are available upon request.

## References

[1] Wymann M.P., Pirola L. (1998). Structure and function of phosphoinositide 3-kinases. Biochim. Biophys. Acta.

[2] Castellano E., Downward J. (2011). RAS Interaction with PI3K: More Than Just Another Effector Pathway. Genes Cancer.

[3] Pacold M.E., Suire S., Perisic O., Lara-Gonzalez S., Davis C.T., Walker E.H., Hawkins P.T., Stephens L., Eccleston J.F., Williams R.L. (2000). Crystal structure and functional analysis of Ras binding to its effector phosphoinositide 3-kinase gamma. Cell.

[4] Rodriguez-Viciana P., Warne P.H., Dhand R., Vanhaesebroeck B., Gout I., Fry M.J., Waterfield M.D., Downward J. (1994). Phosphatidylinositol-3-OH kinase as a direct target of Ras. Nature.

[5] Tolias K.F., Cantley L.C., Carpenter C.L. (1995). Rho family GTPases bind to phosphoinositide kinases. J. Biol. Chem..

[6] Fruman D.A., Meyers R.E., Cantley L.C. (1998). Phosphoinositide kinases. Annu. Rev. Biochem..

[7] Han F., Li C.F., Cai Z., Zhang X., Jin G., Zhang W.N., Xu C., Wang C.Y., Morrow J., Zhang S. (2018). The critical role of AMPK in driving Akt activation under stress, tumorigenesis and drug resistance. Nat. Commun..

[8] Mukhopadhyay S., Chatterjee A., Kogan D., Patel D., Foster D.A. (2015). 5-Aminoimidazole-4-carboxamide-1-beta-4-ribofuranoside (AICAR) enhances the efficacy of rapamycin in human cancer cells. Cell Cycle.

[9] Worby C.A., Dixon J.E. (2014). Pten. Annu. Rev. Biochem..

[10] Burke J.E. (2018). Structural Basis for Regulation of Phosphoinositide Kinases and Their Involvement in Human Disease. Mol. Cell.

[11] Zadra G., Batista J.L., Loda M. (2015). Dissecting the Dual Role of AMPK in Cancer: From Experimental to Human Studies. Mol. Cancer Res..

[12] Delma M.I. (2018). Three May Be Better Than Two: A Proposal for Metformin Addition to PI3K/Akt Inhibitor-antiandrogen Combination in Castration-resistant Prostate Cancer. Cureus.

[13] Domin J., Waterfield M.D. (1997). Using structure to define the function of phosphoinositide 3-kinase family members. FEBS Lett..

[14] Schu P.V., Takegawa K., Fry M.J., Stack J.H., Waterfield M.D., Emr S.D. (1993). Phosphatidylinositol 3-kinase encoded by yeast VPS34 gene essential for protein sorting. Science.

[15] Heng E.Y.Z., Maffucci T. (2022). An Overview of Class II Phosphoinositide 3-Kinases. Curr. Top. Microbiol. Immunol..

[16] Caux M., Chicanne G., Severin S. (2022). Class III PI3K Biology. Curr. Top. Microbiol. Immunol..

[17] Whitman M., Kaplan D.R., Schaffhausen B., Cantley L., Roberts T.M. (1985). Association of phosphatidylinositol kinase activity with polyoma middle-T competent for transformation. Nature.

[18] Escobedo J.A., Navankasattusas S., Kavanaugh W.M., Milfay D., Fried V.A., Williams L.T. (1991). cDNA cloning of a novel 85 kd protein that has SH2 domains and regulates binding of PI3-kinase to the PDGF beta-receptor. Cell.

[19] Otsu M., Hiles I., Gout I., Fry M.J., Ruiz-Larrea F., Panayotou G., Thompson A., Dhand R., Hsuan J., Totty N. (1991). Characterization of two 85 kd proteins that associate with receptor tyrosine kinases, middle-T/pp60c-src complexes, and PI3-kinase. Cell.

[20] Hiles I.D., Otsu M., Volinia S., Fry M.J., Gout I., Dhand R., Panayotou G., Ruiz-Larrea F., Thompson A., Totty N.F. (1992). Phosphatidylinositol 3-kinase: Structure and expression of the 110 kd catalytic subunit. Cell.

[21] Volinia S., Hiles I., Ormondroyd E., Nizetic D., Antonacci R., Rocchi M., Waterfield M.D. (1994). Molecular cloning, cDNA sequence, and chromosomal localization of the human phosphatidylinositol 3-kinase p110 alpha (PIK3CA) gene. Genomics.

[22] Hu P., Mondino A., Skolnik E.Y., Schlessinger J. (1993). Cloning of a novel, ubiquitously expressed human phosphatidylinositol 3-kinase and identification of its binding site on p85. Mol. Cell Biol..

[23] Vanhaesebroeck B., Welham M.J., Kotani K., Stein R., Warne P.H., Zvelebil M.J., Higashi K., Volinia S., Downward J., Waterfield M.D. (1997). P110delta, a novel phosphoinositide 3-kinase in leukocytes. Proc. Natl. Acad. Sci. USA.

[24] Stoyanov B., Volinia S., Hanck T., Rubio I., Loubtchenkov M., Malek D., Stoyanova S., Vanhaesebroeck B., Dhand R., Nurnberg B. (1995). Cloning and characterization of a G protein-activated human phosphoinositide-3 kinase. Science.

[25] Dey B.R., Furlanetto R.W., Nissley S.P. (1998). Cloning of human p55 gamma, a regulatory subunit of phosphatidylinositol 3-kinase, by a yeast two-hybrid library screen with the insulin-like growth factor-I receptor. Gene.

[26] Stephens L.R., Eguinoa A., Erdjument-Bromage H., Lui M., Cooke F., Coadwell J., Smrcka A.S., Thelen M., Cadwallader K., Tempst P. (1997). The G beta gamma sensitivity of a PI3K is dependent upon a tightly associated adaptor, p101. Cell.

[27] Skolnik E.Y., Margolis B., Mohammadi M., Lowenstein E., Fischer R., Drepps A., Ullrich A., Schlessinger J. (1991). Cloning of PI3 kinase-associated p85 utilizing a novel method for expression/cloning of target proteins for receptor tyrosine kinases. Cell.

[28] Carpenter C.L., Duckworth B.C., Auger K.R., Cohen B., Schaffhausen B.S., Cantley L.C. (1990). Purification and characterization of phosphoinositide 3-kinase from rat liver. J. Biol. Chem..

[29] Morgan S.J., Smith A.D., Parker P.J. (1990). Purification and characterization of bovine brain type I phosphatidylinositol kinase. Eur. J. Biochem..

[30] Pons S., Asano T., Glasheen E., Miralpeix M., Zhang Y., Fisher T.L., Myers M.G., Sun X.J., White M.F. (1995). The structure and function of p55PIK reveal a new regulatory subunit for phosphatidylinositol 3-kinase. Mol. Cell Biol..

[31] Inukai K., Funaki M., Ogihara T., Katagiri H., Kanda A., Anai M., Fukushima Y., Hosaka T., Suzuki M., Shin B.C. (1997). p85alpha gene generates three isoforms of regulatory subunit for phosphatidylinositol 3-kinase (PI 3-Kinase), p50alpha, p55alpha, and p85alpha, with different PI 3-kinase activity elevating responses to insulin. J. Biol. Chem..

[32] Fruman D.A., Cantley L.C., Carpenter C.L. (1996). Structural organization and alternative splicing of the murine phosphoinositide 3-kinase p85 alpha gene. Genomics.

[33] Geering B., Cutillas P.R., Nock G., Gharbi S.I., Vanhaesebroeck B. (2007). Class IA phosphoinositide 3-kinases are obligate p85-p110 heterodimers. Proc. Natl. Acad. Sci. USA.

[34] Suire S., Coadwell J., Ferguson G.J., Davidson K., Hawkins P., Stephens L. (2005). p84, a new Gbetagamma-activated regulatory subunit of the type IB phosphoinositide 3-kinase p110gamma. Curr. Biol..

[35] Carpenter C.L., Auger K.R., Chanudhuri M., Yoakim M., Schaffhausen B., Shoelson S., Cantley L.C. (1993). Phosphoinositide 3-kinase is activated by phosphopeptides that bind to the SH2 domains of the 85-kDa subunit. J. Biol. Chem..

[36] Dhand R., Hara K., Hiles I., Bax B., Gout I., Panayotou G., Fry M.J., Yonezawa K., Kasuga M., Waterfield M.D. (1994). PI 3-kinase: Structural and functional analysis of intersubunit interactions. EMBO J..

[37] Stoyanova S., Bulgarelli-Leva G., Kirsch C., Hanck T., Klinger R., Wetzker R., Wymann M.P. (1997). Lipid kinase and protein kinase activities of G-protein-coupled phosphoinositide 3-kinase gamma: Structure-activity analysis and interactions with wortmannin. Biochem. J..

[38] Yu J., Zhang Y., McIlroy J., Rordorf-Nikolic T., Orr G.A., Backer J.M. (1998). Regulation of the p85/p110 phosphatidylinositol 3′-kinase: Stabilization and inhibition of the p110alpha catalytic subunit by the p85 regulatory subunit. Mol. Cell Biol..

[39] Woscholski R., Dhand R., Fry M.J., Waterfield M.D., Parker P.J. (1994). Biochemical characterization of the free catalytic p110 alpha and the complexed heterodimeric p110 alpha.p85 alpha forms of the mammalian phosphatidylinositol 3-kinase. J. Biol. Chem..

[40] Shymanets A., Prajwal, Bucher K., Beer-Hammer S., Harteneck C., Nurnberg B. (2013). p87 and p101 subunits are distinct regulators determining class IB phosphoinositide 3-kinase (PI3K) specificity. J. Biol. Chem..

[41] Klippel A., Escobedo J.A., Hirano M., Williams L.T. (1994). The interaction of small domains between the subunits of phosphatidylinositol 3-kinase determines enzyme activity. Mol. Cell Biol..

[42] Klippel A., Reinhard C., Kavanaugh W.M., Apell G., Escobedo M.A., Williams L.T. (1996). Membrane localization of phosphatidylinositol 3-kinase is sufficient to activate multiple signal-transducing kinase pathways. Mol. Cell Biol..

[43] Booker G.W., Breeze A.L., Downing A.K., Panayotou G., Gout I., Waterfield M.D., Campbell I.D. (1992). Structure of an SH2 domain of the p85 alpha subunit of phosphatidylinositol-3-OH kinase. Nature.

[44] Booker G.W., Gout I., Downing A.K., Driscoll P.C., Boyd J., Waterfield M.D., Campbell I.D. (1993). Solution structure and ligand-binding site of the SH3 domain of the p85 alpha subunit of phosphatidylinositol 3-kinase. Cell.

[45] Koyama S., Yu H., Dalgarno D.C., Shin T.B., Zydowsky L.D., Schreiber S.L. (1993). Structure of the PI3K SH3 domain and analysis of the SH3 family. Cell.

[46] Songyang Z., Shoelson S.E., Chaudhuri M., Gish G., Pawson T., Haser W.G., King F., Roberts T., Ratnofsky S., Lechleider R.J. (1993). SH2 domains recognize specific phosphopeptide sequences. Cell.

[47] Liang J., Chen J.K., Schreiber S.T., Clardy J. (1996). Crystal structure of P13K SH3 domain at 20 angstroms resolution. J. Mol. Biol..

[48] Nolte R.T., Eck M.J., Schlessinger J., Shoelson S.E., Harrison S.C. (1996). Crystal structure of the PI 3-kinase p85 amino-terminal SH2 domain and its phosphopeptide complexes. Nat. Struct. Mol. Biol..

[49] Siegal G., Davis B., Kristensen S.M., Sankar A., Linacre J., Stein R.C., Panayotou G., Waterfield M.D., Driscoll P.C. (1998). Solution structure of the C-terminal SH2 domain of the p85 alpha regulatory subunit of phosphoinositide 3-kinase. J. Mol. Biol..

[50] Yu J., Wjasow C., Backer J.M. (1998). Regulation of the p85/p110alpha phosphatidylinositol 3′-kinase. Distinct roles for the n-terminal and c-terminal SH2 domains. J. Biol. Chem..

[51] Walker E.H., Perisic O., Ried C., Stephens L., Williams R.L. (1999). Structural insights into phosphoinositide 3-kinase catalysis and signalling. Nature.

[52] Fu Z., Aronoff-Spencer E., Wu H., Gerfen G.J., Backer J.M. (2004). The iSH2 domain of PI 3-kinase is a rigid tether for p110 and not a conformational switch. Arch. Biochem. Biophys..

[53] Wu H., Shekar S.C., Flinn R.J., El-Sibai M., Jaiswal B.S., Sen K.I., Janakiraman V., Seshagiri S., Gerfen G.J., Girvin M.E. (2009). Regulation of Class IA PI 3-kinases: C2 domain-iSH2 domain contacts inhibit p85/p110alpha and are disrupted in oncogenic p85 mutants. Proc. Natl. Acad. Sci. USA.

[54] Miller M.S., Schmidt-Kittler O., Bolduc D.M., Brower E.T., Chaves-Moreira D., Allaire M., Kinzler K.W., Jennings I.G., Thompson P.E., Cole P.A. (2014). Structural basis of nSH2 regulation and lipid binding in PI3Kalpha. Oncotarget.

[55] Musacchio A., Cantley L.C., Harrison S.C. (1996). Crystal structure of the breakpoint cluster region-homology domain from phosphoinositide 3-kinase p85 alpha subunit. Proc. Natl. Acad. Sci. USA.

[56] Miled N., Yan Y., Hon W.C., Perisic O., Zvelebil M., Inbar Y., Schneidman-Duhovny D., Wolfson H.J., Backer J.M., Williams R.L. (2007). Mechanism of two classes of cancer mutations in the phosphoinositide 3-kinase catalytic subunit. Science.

[57] Zhang X., Vadas O., Perisic O., Anderson K.E., Clark J., Hawkins P.T., Stephens L.R., Williams R.L. (2011). Structure of lipid kinase p110beta/p85beta elucidates an unusual SH2-domain-mediated inhibitory mechanism. Mol. Cell.

[58] Huang C.H., Mandelker D., Schmidt-Kittler O., Samuels Y., Velculescu V.E., Kinzler K.W., Vogelstein B., Gabelli S.B., Amzel L.M. (2007). The structure of a human p110alpha/p85alpha complex elucidates the effects of oncogenic PI3Kalpha mutations. Science.

[59] Chen C.L., Syahirah R., Ravala S.K., Yen Y.C., Klose T., Deng Q., Tesmer J.J.G. (2024). Molecular basis for Gbetagamma-mediated activation of phosphoinositide 3-kinase gamma. Nat. Struct. Mol. Biol..

[60] Vogt P.K., Hart J.R., Yang S., Zhou Q., Yang D., Wang M.W. (2023). Structural and mechanistic insights provided by single particle cryo-EM analysis of phosphoinositide 3-kinase (PI3Kalpha). Biochim. Biophys. Acta Rev. Cancer.

[61] Zhou Q., Liu X., Neri D., Li W., Favalli N., Bassi G., Yang S., Yang D., Vogt P.K., Wang M.W. (2023). Structural insights into the interaction of three Y-shaped ligands with PI3Kalpha. Proc. Natl. Acad. Sci. USA.

[62] Rathinaswamy M.K., Dalwadi U., Fleming K.D., Adams C., Stariha J.T.B., Pardon E., Baek M., Vadas O., DiMaio F., Steyaert J. (2021). Structure of the phosphoinositide 3-kinase (PI3K) p110gamma-p101 complex reveals molecular mechanism of GPCR activation. Sci. Adv..

[63] Amzel L.M., Huang C.H., Mandelker D., Lengauer C., Gabelli S.B., Vogelstein B. (2008). Structural comparisons of class I phosphoinositide 3-kinases. Nat. Rev. Cancer.

[64] Walker E.H., Pacold M.E., Perisic O., Stephens L., Hawkins P.T., Wymann M.P., Williams R.L. (2000). Structural determinants of phosphoinositide 3-kinase inhibition by wortmannin, LY294002, quercetin, myricetin, and staurosporine. Mol. Cell.

[65] Berndt A., Miller S., Williams O., Le D.D., Houseman B.T., Pacold J.I., Gorrec F., Hon W.C., Liu Y., Rommel C. (2010). The p110 delta structure: Mechanisms for selectivity and potency of new PI(3)K inhibitors. Nat. Chem. Biol..

[66] Jumper J., Evans R., Pritzel A., Green T., Figurnov M., Ronneberger O., Tunyasuvunakool K., Bates R., Zidek A., Potapenko A. (2021). Highly accurate protein structure prediction with AlphaFold. Nature.

[67] Zhao Y., Zhang X., Chen Y., Lu S., Peng Y., Wang X., Guo C., Zhou A., Zhang J., Luo Y. (2014). Crystal Structures of PI3Kalpha Complexed with PI103 and Its Derivatives: New Directions for Inhibitors Design. ACS Med. Chem. Lett..

[68] Mandelker D., Gabelli S.B., Schmidt-Kittler O., Zhu J., Cheong I., Huang C.H., Kinzler K.W., Vogelstein B., Amzel L.M. (2009). A frequent kinase domain mutation that changes the interaction between PI3Kalpha and the membrane. Proc. Natl. Acad. Sci. USA.

[69] Yang H.W., Shin M.G., Lee S., Kim J.R., Park W.S., Cho K.H., Meyer T., Heo W.D. (2012). Cooperative activation of PI3K by Ras and Rho family small GTPases. Mol. Cell.

[70] Whitecross D.E., Anderson D.H. (2017). Identification of the Binding Sites on Rab5 and p110beta Phosphatidylinositol 3-kinase. Sci. Rep..

[71] Zhang M., Jang H., Nussinov R. (2019). The structural basis for Ras activation of PI3Kalpha lipid kinase. Phys. Chem. Chem. Phys..

[72] Fukushima S., Matsuoka S., Ueda M. (2019). Excitable dynamics of Ras triggers spontaneous symmetry breaking of PIP3 signaling in motile cells. J. Cell Sci..

[73] Huang L., Hofer F., Martin G.S., Kim S.H. (1998). Structural basis for the interaction of Ras with RalGDS. Nat. Struct. Mol. Biol..

[74] Nassar N., Horn G., Herrmann C., Scherer A., McCormick F., Wittinghofer A. (1995). The 2.2 A crystal structure of the Ras-binding domain of the serine/threonine kinase c-Raf1 in complex with Rap1A and a GTP analogue. Nature.

[75] Corbalan-Garcia S., Gomez-Fernandez J.C. (2014). Signaling through C2 domains: More than one lipid target. Biochim. Biophys. Acta.

[76] Thapa N., Chen M., Cryns V.L., Anderson R. (2024). A p85 isoform switch enhances PI3K activation on endosomes by a MAP4- and PI3P-dependent mechanism. Cell Rep..

[77] Gabelli S.B., Mandelker D., Schmidt-Kittler O., Vogelstein B., Amzel L.M. (2010). Somatic mutations in PI3Kalpha: Structural basis for enzyme activation and drug design. Biochim. Biophys. Acta.

[78] Huse M., Kuriyan J. (2002). The conformational plasticity of protein kinases. Cell.

[79] Vanhaesebroeck B., Perry M.W.D., Brown J.R., Andre F., Okkenhaug K. (2021). PI3K inhibitors are finally coming of age. Nat. Rev. Drug Discov..

[80] Maheshwari S., Miller M.S., O’Meally R., Cole R.N., Amzel L.M., Gabelli S.B. (2017). Kinetic and structural analyses reveal residues in phosphoinositide 3-kinase alpha that are critical for catalysis and substrate recognition. J. Biol. Chem..

[81] Hoedemaeker F.J., Siegal G., Roe S.M., Driscoll P.C., Abrahams J.P. (1999). Crystal structure of the C-terminal SH2 domain of the p85alpha regulatory subunit of phosphoinositide 3-kinase: An SH2 domain mimicking its own substrate. J. Mol. Biol..

[82] Dombrosky-Ferlan P.M., Corey S.J. (1997). Yeast two-hybrid in vivo association of the Src kinase Lyn with the proto-oncogene product Cbl but not with the p85 subunit of PI 3-kinase. Oncogene.

[83] Diekmann D., Brill S., Garrett M.D., Totty N., Hsuan J., Monfries C., Hall C., Lim L., Hall A. (1991). Bcr encodes a GTPase-activating protein for p21rac. Nature.

[84] Holt K.H., Olson L., Moye-Rowley W.S., Pessin J.E. (1994). Phosphatidylinositol 3-kinase activation is mediated by high-affinity interactions between distinct domains within the p110 and p85 subunits. Mol. Cell Biol..

[85] Hale B.G., Kerry P.S., Jackson D., Precious B.L., Gray A., Killip M.J., Randall R.E., Russell R.J. (2010). Structural insights into phosphoinositide 3-kinase activation by the influenza A virus NS1 protein. Proc. Natl. Acad. Sci. USA.

[86] Voigt P., Brock C., Nurnberg B., Schaefer M. (2005). Assigning functional domains within the p101 regulatory subunit of phosphoinositide 3-kinase gamma. J. Biol. Chem..

[87] Hoxhaj G., Manning B.D. (2020). The PI3K-AKT network at the interface of oncogenic signalling and cancer metabolism. Nat. Rev. Cancer.

[88] Rommel C., Camps M., Ji H. (2007). PI3K delta and PI3K gamma: Partners in crime in inflammation in rheumatoid arthritis and beyond?. Nat. Rev. Immunol..

[89] Park S.J., Min K.H., Lee Y.C. (2008). Phosphoinositide 3-kinase delta inhibitor as a novel therapeutic agent in asthma. Respirology.

[90] Bi L., Okabe I., Bernard D.J., Nussbaum R.L. (2002). Early embryonic lethality in mice deficient in the p110beta catalytic subunit of PI 3-kinase. Mamm. Genome.

[91] Ciraolo E., Iezzi M., Marone R., Marengo S., Curcio C., Costa C., Azzolino O., Gonella C., Rubinetto C., Wu H. (2008). Phosphoinositide 3-kinase p110beta activity: Key role in metabolism and mammary gland cancer but not development. Sci. Signal..

[92] Foukas L.C., Claret M., Pearce W., Okkenhaug K., Meek S., Peskett E., Sancho S., Smith A.J., Withers D.J., Vanhaesebroeck B. (2006). Critical role for the p110alpha phosphoinositide-3-OH kinase in growth and metabolic regulation. Nature.

[93] Graupera M., Guillermet-Guibert J., Foukas L.C., Phng L.K., Cain R.J., Salpekar A., Pearce W., Meek S., Millan J., Cutillas P.R. (2008). Angiogenesis selectively requires the p110alpha isoform of PI3K to control endothelial cell migration. Nature.

[94] Sopasakis V.R., Liu P., Suzuki R., Kondo T., Winnay J., Tran T.T., Asano T., Smyth G., Sajan M.P., Farese R.V. (2010). Specific roles of the p110alpha isoform of phosphatidylinsositol 3-kinase in hepatic insulin signaling and metabolic regulation. Cell Metab..

[95] Guillermet-Guibert J., Bjorklof K., Salpekar A., Gonella C., Ramadani F., Bilancio A., Meek S., Smith A.J., Okkenhaug K., Vanhaesebroeck B. (2008). The p110beta isoform of phosphoinositide 3-kinase signals downstream of G protein-coupled receptors and is functionally redundant with p110gamma. Proc. Natl. Acad. Sci. USA.

[96] Jia S., Liu Z., Zhang S., Liu P., Zhang L., Lee S.H., Zhang J., Signoretti S., Loda M., Roberts T.M. (2008). Essential roles of PI(3)K-p110beta in cell growth, metabolism and tumorigenesis. Nature.

[97] Ando Y., Iwasa S., Takahashi S., Saka H., Kakizume T., Natsume K., Suenaga N., Quadt C., Yamada Y. (2019). Phase I study of alpelisib (BYL719), an alpha-specific PI3K inhibitor, in Japanese patients with advanced solid tumors. Cancer Sci..

[98] Martin V., Guillermet-Guibert J., Chicanne G., Cabou C., Jandrot-Perrus M., Plantavid M., Vanhaesebroeck B., Payrastre B., Gratacap M.P. (2010). Deletion of the p110beta isoform of phosphoinositide 3-kinase in platelets reveals its central role in Akt activation and thrombus formation in vitro and in vivo. Blood.

[99] Marques M., Kumar A., Poveda A.M., Zuluaga S., Hernandez C., Jackson S., Pasero P., Carrera A.C. (2009). Specific function of phosphoinositide 3-kinase beta in the control of DNA replication. Proc. Natl. Acad. Sci. USA.

[100] Ciraolo E., Morello F., Hobbs R.M., Wolf F., Marone R., Iezzi M., Lu X., Mengozzi G., Altruda F., Sorba G. (2010). Essential role of the p110beta subunit of phosphoinositide 3-OH kinase in male fertility. Mol. Biol. Cell.

[101] Thorpe L.M., Yuzugullu H., Zhao J.J. (2015). PI3K in cancer: Divergent roles of isoforms, modes of activation and therapeutic targeting. Nat. Rev. Cancer.

[102] Bi L., Okabe I., Bernard D.J., Wynshaw-Boris A., Nussbaum R.L. (1999). Proliferative defect and embryonic lethality in mice homozygous for a deletion in the p110alpha subunit of phosphoinositide 3-kinase. J. Biol. Chem..

[103] Samuels Y., Wang Z., Bardelli A., Silliman N., Ptak J., Szabo S., Yan H., Gazdar A., Powell S.M., Riggins G.J. (2004). High frequency of mutations of the PIK3CA gene in human cancers. Science.

[104] Lindhurst M.J., Parker V.E., Payne F., Sapp J.C., Rudge S., Harris J., Witkowski A.M., Zhang Q., Groeneveld M.P., Scott C.E. (2012). Mosaic overgrowth with fibroadipose hyperplasia is caused by somatic activating mutations in PIK3CA. Nat. Genet..

[105] Arafeh R., Samuels Y. (2019). PIK3CA in cancer: The past 30 years. Semin. Cancer Biol..

[106] Buckbinder L., St Jean D.J., Tieu T., Ladd B., Hilbert B., Wang W., Alltucker J.T., Manimala S., Kryukov G.V., Brooijmans N. (2023). STX-478, a Mutant-Selective, Allosteric PI3Kalpha Inhibitor Spares Metabolic Dysfunction and Improves Therapeutic Response in PI3Kalpha-Mutant Xenografts. Cancer Discov..

[107] Vanhaesebroeck B., Burke J.E., Madsen R.R. (2022). Precision Targeting of Mutant PI3Kalpha in Cancer by Selective Degradation. Cancer Discov..

[108] Nakanishi Y., Walter K., Spoerke J.M., O’Brien C., Huw L.Y., Hampton G.M., Lackner M.R. (2016). Activating Mutations in PIK3CB Confer Resistance to PI3K Inhibition and Define a Novel Oncogenic Role for p110beta. Cancer Res..

[109] Whale A.D., Colman L., Lensun L., Rogers H.L., Shuttleworth S.J. (2017). Functional characterization of a novel somatic oncogenic mutation of PIK3CB. Signal Transduct. Target. Ther..

[110] Pazarentzos E., Giannikopoulos P., Hrustanovic G., St John J., Olivas V.R., Gubens M.A., Balassanian R., Weissman J., Polkinghorn W., Bivona T.G. (2016). Oncogenic activation of the PI3-kinase p110beta isoform via the tumor-derived PIK3Cbeta(D1067V) kinase domain mutation. Oncogene.

[111] Lucas C.L., Chandra A., Nejentsev S., Condliffe A.M., Okkenhaug K. (2016). PI3Kdelta and primary immunodeficiencies. Nat. Rev. Immunol..

[112] Durandy A., Kracker S. (2020). Increased activation of PI3 kinase-delta predisposes to B-cell lymphoma. Blood.

[113] Thian M., Hoeger B., Kamnev A., Poyer F., Kostel Bal S., Caldera M., Jimenez-Heredia R., Huemer J., Pickl W.F., Gross M. (2020). Germline biallelic PIK3CG mutations in a multifaceted immunodeficiency with immune dysregulation. Haematologica.

[114] Sasaki T., Irie-Sasaki J., Horie Y., Bachmaier K., Fata J.E., Li M., Suzuki A., Bouchard D., Ho A., Redston M. (2000). Colorectal carcinomas in mice lacking the catalytic subunit of PI(3)Kgamma. Nature.

[115] Urick M.E., Rudd M.L., Godwin A.K., Sgroi D., Merino M., Bell D.W. (2011). PIK3R1 (p85alpha) is somatically mutated at high frequency in primary endometrial cancer. Cancer Res..

[116] Hofmann B.T., Jucker M. (2012). Activation of PI3K/Akt signaling by n-terminal SH2 domain mutants of the p85alpha regulatory subunit of PI3K is enhanced by deletion of its c-terminal SH2 domain. Cell Signal..

[117] Deau M.C., Heurtier L., Frange P., Suarez F., Bole-Feysot C., Nitschke P., Cavazzana M., Picard C., Durandy A., Fischer A. (2014). A human immunodeficiency caused by mutations in the PIK3R1 gene. J. Clin. Investig..

[118] Thorpe L.M., Spangle J.M., Ohlson C.E., Cheng H., Roberts T.M., Cantley L.C., Zhao J.J. (2017). PI3K-p110alpha mediates the oncogenic activity induced by loss of the novel tumor suppressor PI3K-p85alpha. Proc. Natl. Acad. Sci. USA.

[119] Fox M., Mott H.R., Owen D. (2020). Class IA PI3K regulatory subunits: p110-independent roles and structures. Biochem. Soc. Trans..

[120] Cheung L.W., Mills G.B. (2016). Targeting therapeutic liabilities engendered by PIK3R1 mutations for cancer treatment. Pharmacogenomics.

[121] Gabelli S.B., Huang C.H., Mandelker D., Schmidt-Kittler O., Vogelstein B., Amzel L.M. (2010). Structural effects of oncogenic PI3Kalpha mutations. Curr. Top. Microbiol. Immunol..

[122] Gymnopoulos M., Elsliger M.A., Vogt P.K. (2007). Rare cancer-specific mutations in PIK3CA show gain of function. Proc. Natl. Acad. Sci. USA.

[123] Rudd M.L., Price J.C., Fogoros S., Godwin A.K., Sgroi D.C., Merino M.J., Bell D.W. (2011). A unique spectrum of somatic PIK3CA (p110alpha) mutations within primary endometrial carcinomas. Clin. Cancer Res..

[124] Zhao L., Vogt P.K. (2008). Class I PI3K in oncogenic cellular transformation. Oncogene.

[125] Huang X., Wang K., Han J., Chen X., Wang Z., Wu T., Yu B., Zhao F., Wang X., Li H. (2024). Cryo-EM structures reveal two allosteric inhibition modes of PI3Kalpha(H1047R) involving a re-shaping of the activation loop. Structure.

[126] Vasan N., Razavi P., Johnson J.L., Shao H., Shah H., Antoine A., Ladewig E., Gorelick A., Lin T.Y., Toska E. (2019). Double PIK3CA mutations in cis increase oncogenicity and sensitivity to PI3Kalpha inhibitors. Science.

[127] Liu X., Zhou Q., Hart J.R., Xu Y., Yang S., Yang D., Vogt P.K., Wang M.W. (2022). Cryo-EM structures of cancer-specific helical and kinase domain mutations of PI3Kalpha. Proc. Natl. Acad. Sci. USA.

[128] Debouki-Joudi S., Ben Kridis W., Trifa F., Ayadi W., Khabir A., Sellami-Boudawara T., Daoud J., Khanfir A., Mokdad-Gargouri R. (2024). A novel PIK3CA hot-spot mutation in breast cancer patients detected by HRM-COLD-PCR analysis. Breast Dis..

[129] Spangle J.M., Von T., Pavlick D.C., Khotimsky A., Zhao J.J., Roberts T.M. (2020). PIK3CA C-terminal frameshift mutations are novel oncogenic events that sensitize tumors to PI3K-alpha inhibition. Proc. Natl. Acad. Sci. USA.

[130] Kandoth C., McLellan M.D., Vandin F., Ye K., Niu B., Lu C., Xie M., Zhang Q., McMichael J.F., Wyczalkowski M.A. (2013). Mutational landscape and significance across 12 major cancer types. Nature.

[131] Dsouza N.R., Cottrell C.E., Davies O.M.T., Tollefson M.M., Frieden I.J., Basel D., Urrutia R., Drolet B.A., Zimmermann M.T. (2024). Structural and Dynamic Analyses of Pathogenic Variants in PIK3R1 Reveal a Shared Mechanism Associated among Cancer, Undergrowth, and Overgrowth Syndromes. Life.

[132] Sun M., Hillmann P., Hofmann B.T., Hart J.R., Vogt P.K. (2010). Cancer-derived mutations in the regulatory subunit p85alpha of phosphoinositide 3-kinase function through the catalytic subunit p110alpha. Proc. Natl. Acad. Sci. USA.

[133] Jimenez C., Jones D.R., Rodriguez-Viciana P., Gonzalez-Garcia A., Leonardo E., Wennstrom S., von Kobbe C., Toran J.L., R-Borlado L., Calvo V. (1998). Identification and characterization of a new oncogene derived from the regulatory subunit of phosphoinositide 3-kinase. EMBO J..

[134] Hao Y., He B., Wu L., Li Y., Wang C., Wang T., Sun L., Zhang Y., Zhan Y., Zhao Y. (2022). Nuclear translocation of p85beta promotes tumorigenesis of PIK3CA helical domain mutant cancer. Nat. Commun..

[135] Jaiswal B.S., Janakiraman V., Kljavin N.M., Chaudhuri S., Stern H.M., Wang W., Kan Z., Dbouk H.A., Peters B.A., Waring P. (2009). Somatic mutations in p85alpha promote tumorigenesis through class IA PI3K activation. Cancer Cell.

[136] Liu S., Knapp S., Ahmed A.A. (2014). The structural basis of PI3K cancer mutations: From mechanism to therapy. Cancer Res..

[137] Cheung L.W., Yu S., Zhang D., Li J., Ng P.K., Panupinthu N., Mitra S., Ju Z., Yu Q., Liang H. (2014). Naturally occurring neomorphic PIK3R1 mutations activate the MAPK pathway, dictating therapeutic response to MAPK pathway inhibitors. Cancer Cell.

[138] Tsolakos N., Durrant T.N., Chessa T., Suire S.M., Oxley D., Kulkarni S., Downward J., Perisic O., Williams R.L., Stephens L. (2018). Quantitation of class IA PI3Ks in mice reveals p110-free-p85s and isoform-selective subunit associations and recruitment to receptors. Proc. Natl. Acad. Sci. USA.

[139] Skorski T., Bellacosa A., Nieborowska-Skorska M., Majewski M., Martinez R., Choi J.K., Trotta R., Wlodarski P., Perrotti D., Chan T.O. (1997). Transformation of hematopoietic cells by BCR/ABL requires activation of a PI-3k/Akt-dependent pathway. EMBO J..

[140] Gupta S., Ramjaun A.R., Haiko P., Wang Y., Warne P.H., Nicke B., Nye E., Stamp G., Alitalo K., Downward J. (2007). Binding of ras to phosphoinositide 3-kinase p110alpha is required for ras-driven tumorigenesis in mice. Cell.

[141] Wu C.Y., Carpenter E.S., Takeuchi K.K., Halbrook C.J., Peverley L.V., Bien H., Hall J.C., DelGiorno K.E., Pal D., Song Y. (2014). PI3K regulation of RAC1 is required for KRAS-induced pancreatic tumorigenesis in mice. Gastroenterology.

[142] Kang S., Denley A., Vanhaesebroeck B., Vogt P.K. (2006). Oncogenic transformation induced by the p110beta, -gamma, and -delta isoforms of class I phosphoinositide 3-kinase. Proc. Natl. Acad. Sci. USA.

[143] Suire S., Condliffe A.M., Ferguson G.J., Ellson C.D., Guillou H., Davidson K., Welch H., Coadwell J., Turner M., Chilvers E.R. (2006). Gbetagammas and the Ras binding domain of p110gamma are both important regulators of PI(3)Kgamma signalling in neutrophils. Nat. Cell Biol..

[144] Zhao J.J., Liu Z., Wang L., Shin E., Loda M.F., Roberts T.M. (2005). The oncogenic properties of mutant p110alpha and p110beta phosphatidylinositol 3-kinases in human mammary epithelial cells. Proc. Natl. Acad. Sci. USA.

[145] Denley A., Kang S., Karst U., Vogt P.K. (2008). Oncogenic signaling of class I PI3K isoforms. Oncogene.

[146] Dbouk H.A., Pang H., Fiser A., Backer J.M. (2010). A biochemical mechanism for the oncogenic potential of the p110beta catalytic subunit of phosphoinositide 3-kinase. Proc. Natl. Acad. Sci. USA.

[147] Maier U., Babich A., Nurnberg B. (1999). Roles of non-catalytic subunits in gbetagamma-induced activation of class I phosphoinositide 3-kinase isoforms beta and gamma. J. Biol. Chem..

[148] Kurosu H., Maehama T., Okada T., Yamamoto T., Hoshino S., Fukui Y., Ui M., Hazeki O., Katada T. (1997). Heterodimeric phosphoinositide 3-kinase consisting of p85 and p110beta is synergistically activated by the betagamma subunits of G proteins and phosphotyrosyl peptide. J. Biol. Chem..

[149] Foukas L.C., Berenjeno I.M., Gray A., Khwaja A., Vanhaesebroeck B. (2010). Activity of any class IA PI3K isoform can sustain cell proliferation and survival. Proc. Natl. Acad. Sci. USA.

[150] Kulkarni S., Sitaru C., Jakus Z., Anderson K.E., Damoulakis G., Davidson K., Hirose M., Juss J., Oxley D., Chessa T.A. (2011). PI3Kbeta plays a critical role in neutrophil activation by immune complexes. Sci. Signal..

[151] Zhao J.J., Gjoerup O.V., Subramanian R.R., Cheng Y., Chen W., Roberts T.M., Hahn W.C. (2003). Human mammary epithelial cell transformation through the activation of phosphatidylinositol 3-kinase. Cancer Cell.

[152] Kang S., Bader A.G., Vogt P.K. (2005). Phosphatidylinositol 3-kinase mutations identified in human cancer are oncogenic. Proc. Natl. Acad. Sci. USA.

[153] Wee S., Wiederschain D., Maira S.M., Loo A., Miller C., deBeaumont R., Stegmeier F., Yao Y.M., Lengauer C. (2008). PTEN-deficient cancers depend on PIK3CB. Proc. Natl. Acad. Sci. USA.

[154] Pridham K.J., Le L., Guo S., Varghese R.T., Algino S., Liang Y., Fajardin R., Rodgers C.M., Simonds G.R., Kelly D.F. (2018). PIK3CB/p110beta is a selective survival factor for glioblastoma. Neuro Oncol..

[155] Xie S., Ni J., McFaline-Figueroa J.R., Wang Y., Bronson R.T., Ligon K.L., Wen P.Y., Roberts T.M., Zhao J.J. (2020). Divergent Roles of PI3K Isoforms in PTEN-Deficient Glioblastomas. Cell Rep..

[156] Pridham K.J., Hutchings K.R., Beck P., Liu M., Xu E., Saechin E., Bui V., Patel C., Solis J., Huang L. (2024). Selective regulation of chemosensitivity in glioblastoma by phosphatidylinositol 3-kinase beta. iScience.

[157] Pridham K.J., Shah F., Hutchings K.R., Sheng K.L., Guo S., Liu M., Kanabur P., Lamouille S., Lewis G., Morales M. (2022). Connexin 43 confers chemoresistance through activating PI3K. Oncogenesis.

[158] Miller K.A., Degan S., Wang Y., Cohen J., Ku S.Y., Goodrich D.W., Gelman I.H. (2024). PTEN-regulated PI3K-p110 and AKT isoform plasticity controls metastatic prostate cancer progression. Oncogene.

[159] Kumar A., Redondo-Munoz J., Perez-Garcia V., Cortes I., Chagoyen M., Carrera A.C. (2011). Nuclear but not cytosolic phosphoinositide 3-kinase beta has an essential function in cell survival. Mol. Cell Biol..

[160] Kumar A., Fernandez-Capetillo O., Carrera A.C. (2010). Nuclear phosphoinositide 3-kinase beta controls double-strand break DNA repair. Proc. Natl. Acad. Sci. USA.

[161] Dbouk H.A., Vadas O., Shymanets A., Burke J.E., Salamon R.S., Khalil B.D., Barrett M.O., Waldo G.L., Surve C., Hsueh C. (2012). G protein-coupled receptor-mediated activation of p110beta by Gbetagamma is required for cellular transformation and invasiveness. Sci. Signal..

[162] Markham A. (2014). Idelalisib: First global approval. Drugs.

[163] Blair H.A. (2018). Duvelisib: First Global Approval. Drugs.

[164] Dhillon S., Keam S.J. (2021). Umbralisib: First Approval. Drugs.

[165] FDA U.S (2022). Phosphatidylinositol 3-kinase (PI3K) Inhibitors in Hematologic Malgnancies. Oncologic Drugs Advisory Committee Meeting. https://www.fda.gov/media/157762/download.

[166] FDA U.S., Gilead Sciences, Inc. Withdrawal of Approval of Indications for Relapsed Follicular Lymphoma and Relapsed Small Lymphocytic Lymphoma for ZYDELIG (Idelalisib) Tablets. Federal Register, The Daily Journal of the United States Government. 2022: Published Document: 2022–11277 (87 FR 32031). https://www.federalregister.gov/documents/2022/05/26/2022-11277/gilead-sciences-inc-withdrawal-of-approval-of-indications-for-relapsed-follicular-lymphoma-and#:~:text=Therefore%2C%20under%20%C2%A7%20314.150(d,for%20ZYDELIG%20(idelalisib)%20Tablets.

[167] U.S. FDA, Secura Bio, Inc. Withdrawal of Approval of Relapsed or Refractory Follicular Lymphoma Indication for COPIKTRA. Federal Register, The Daily Journal of the United States Government. 2022:Published Document: 2022–07931 (87 FR 21888). https://www.federalregister.gov/documents/2022/04/13/2022-07931/secura-bio-inc-withdrawal-of-approval-of-relapsed-or-refractory-follicular-lymphoma-indication-for#:~:text=Therefore%2C%20under%20%C2%A7%20314.150(d,other%20approved%20indication%20for%20COPIKTRA.

[168] FDA U.S., TG Therapeutics, Inc. Withdrawal of Approval of New Drug Application for UKONIQ (Umbralisib Tosylate) Tablets, Equivalent to 200 Milligrams Base. Federal Register, The Daily Journal of the United States Government. 2022:Published Document: 2022–11631 (87 FR 32425). https://www.federalregister.gov/documents/2022/05/31/2022-11631/tg-therapeutics-inc-withdrawal-of-approval-of-new-drug-application-for-ukoniq-umbralisib-tosylate#:~:text=0503%2DAA39%20SORN-,TG%20Therapeutics%2C%20Inc.%3B%20Withdrawal%20of%20Approval%20of%20New%20Drug,Equivalent%20to%20200%20Milligrams%20Base&text=Approval%20is%20withdrawn%20as%20of%20May%2031%2C%202022.&text=Document%20page%20views%20are%20updated,cumulative%20counts%20for%20this%20document.

[169] Sk A., Hemalatha K., Matada G.S.P., Pal R., Manjushree B., Mounika S., Haripriya E., Viji M., Anjan D. (2024). Current developments in PI3K-based anticancer agents: Designing strategies, biological activity, selectivity, structure-activity correlation, and docking insight. Bioorg. Chem..

[170] Varkaris A., Fece de la Cruz F., Martin E.E., Norden B.L., Chevalier N., Kehlmann A.M., Leshchiner I., Barnes H., Ehnstrom S., Stavridi A.M. (2024). Allosteric PI3Kalpha Inhibition Overcomes On-target Resistance to Orthosteric Inhibitors Mediated by Secondary PIK3CA Mutations. Cancer Discov..

